# The virulence factor LecB varies in clinical isolates: consequences for ligand binding and drug discovery†
†Electronic supplementary information (ESI) available: Multiple sequence alignment of LecB from clinical isolates. Concentration determination of FITC-labeled lectins LecB_PAO1_ and LecB_PA14_. Chemical structure drawing of compound 543. Dynamic light scattering of LecB_PA14_. Titration curves for fluorescence polarization-based direct and competitive binding assay. Titration figures for ITC experiments, as well as for compound **23** MALDI-MS and ^1^H-NMR. Data collection and refinement statistics for LecB_PA14_ structures. See DOI: 10.1039/c6sc00696e


**DOI:** 10.1039/c6sc00696e

**Published:** 2016-05-11

**Authors:** Roman Sommer, Stefanie Wagner, Annabelle Varrot, Corwin M. Nycholat, Ariane Khaledi, Susanne Häussler, James C. Paulson, Anne Imberty, Alexander Titz

**Affiliations:** a Chemical Biology of Carbohydrates , Helmholtz Institute for Pharmaceutical Research Saarland (HIPS) , D-66123 Saarbrücken , Germany . Email: alexander.titz@helmholtz-hzi.de ; http://www.helmholtz-hzi.de/cbch; b Deutsches Zentrum für Infektionsforschung (DZIF) , Standort Hannover , Braunschweig , Germany; c Centre de Recherche sur les Macromolécules Végétales (CERMAV-UPR5301) , CNRS and Université Grenoble Alpes , BP53 , F-38041 Grenoble cedex 9 , France; d Department of Cell and Molecular Biology and Department of Chemical Physiology , The Scripps Research Institute , 10550 North Torrey Pines Road , La Jolla , CA 92037 , USA; e Molecular Bacteriology , Helmholtz Centre for Infection Research , D-38124 Braunschweig , Germany

## Abstract

*P. aeruginosa* causes a substantial number of nosocomial infections and is the leading cause of death of cystic fibrosis patients. This Gram-negative bacterium is highly resistant against antibiotics and further protects itself by forming a biofilm. Moreover, a high genomic variability among clinical isolates complicates therapy. Its lectin LecB is a virulence factor and necessary for adhesion and biofilm formation. We analyzed the sequence of LecB variants in a library of clinical isolates and demonstrate that it can serve as a marker for strain family classification. LecB from the highly virulent model strain PA14 presents 13% sequence divergence with LecB from the well characterized PAO1 strain. These differences might result in differing ligand binding specificities and ultimately in reduced efficacy of drugs directed towards LecB. Despite several amino acid variations at the carbohydrate binding site, glycan array analysis showed a comparable binding pattern for both variants. A common high affinity ligand could be identified and after its chemoenzymatic synthesis verified in a competitive binding assay: an *N*-glycan presenting two blood group O epitopes (H-type 2 antigen). Molecular modeling of the complex suggests a bivalent interaction of the ligand with the LecB tetramer by bridging two separate binding sites. This binding rationalizes the strong avidity (35 nM) of LecB_PA14_ to this human fucosylated N-glycan. Biochemical evaluation of a panel of glycan ligands revealed that LecB_PA14_ demonstrated higher glycan affinity compared to LecB_PAO1_ including the extraordinarily potent affinity of 70 nM towards the monovalent human antigen Lewis^a^. The structural basis of this unusual high affinity ligand binding for lectins was rationalized by solving the protein crystal structures of LecB_PA14_ with several glycans.

## Abstract 2

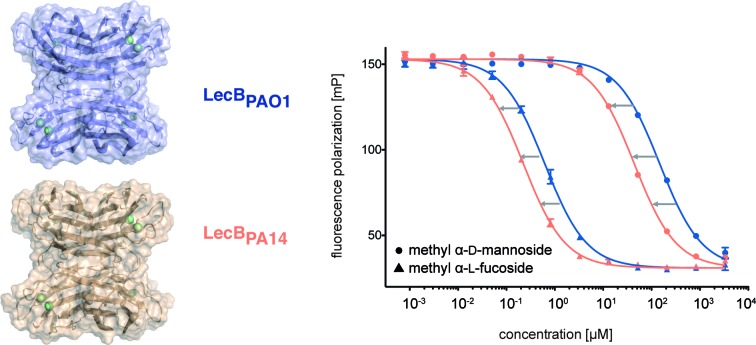
The sequence of the virulence factor LecB differs significantly between the evolutionarily diverged PAO1- or PA14-like strains and can serve as marker for strain classification. Despite these variations, its comparable ligand selectivity makes LecB a highly promising target for anti-virulence therapy.

## Introduction

*Pseudomonas aeruginosa* belongs to the ESKAPE pathogens, which cause a substantial number of nosocomial infections and successfully escape from antimicrobial effects of antibiotic treatments.^1^ The Gram-negative opportunistic pathogen *P. aeruginosa* is a major threat to immunocompromised patients and individuals suffering from cystic fibrosis. *P. aeruginosa* effectively applies a wide variety of defense mechanisms to escape from antimicrobials, *e.g.* genetic adaptation, efflux pumps, virulence factors and biofilm formation.^2^ The physical barrier of the biofilm matrix^3^ protects the bacteria embedded in these social colonies against host defense and antibiotic treatment impeding successful therapy.^4^ Biofilm embedded cells are 10- to 1000-fold more resistant towards a number of antimicrobial drugs than in planktonic culture.^5^ The dramatic increase of antibiotic resistances in the last decades is a central problem and new therapies are urgently needed.^6^ Antivirulence therapies are an alternative strategy that block bacterial virulence without killing or inhibiting bacterial growth and are therefore expected to avoid rapid development of resistance.^7^

**Fig. 1:**
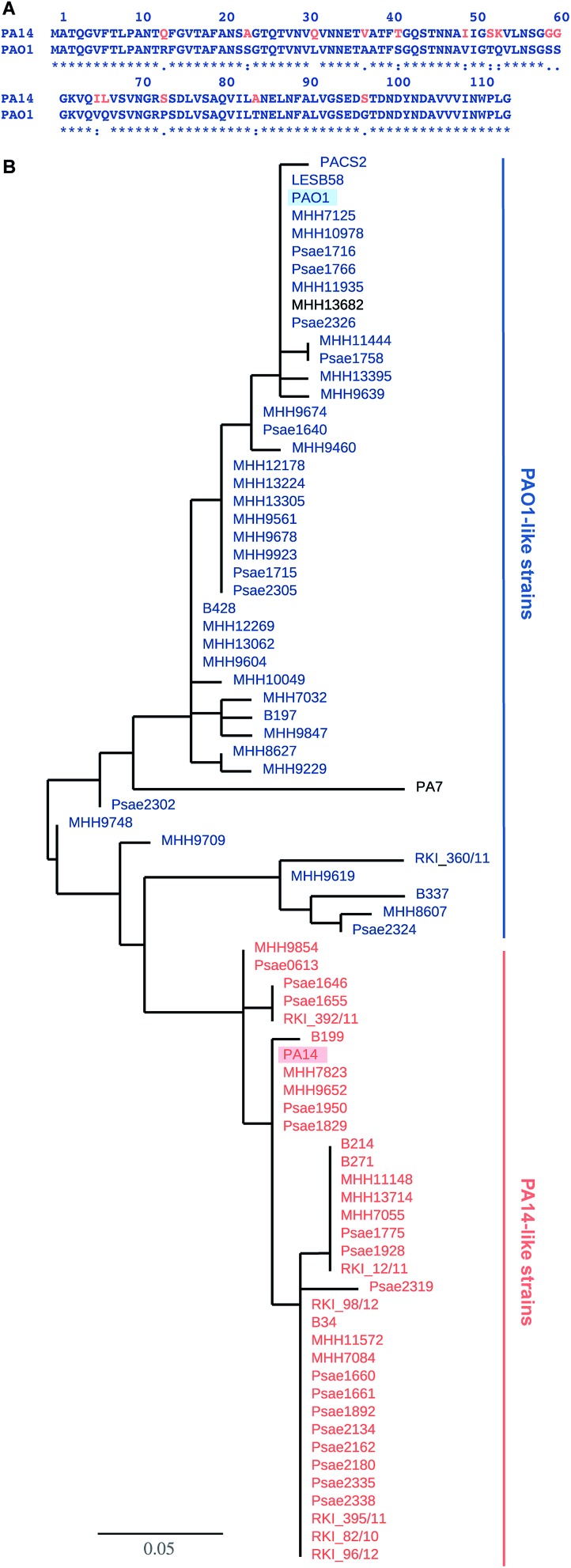
LecB sequence comparison: (A) alignment of LecB sequences from PA14 and PAO1. Amino acid numbering corresponds to the crystal structure of LecB_PAO1_, which lacks methionine; (B) phylogenetic tree based on LecB sequences from *P. aeruginosa* clinical isolates and reference strains PAO1, PA7, PA14, PACS2, LESB58. An independent phylogenetic analysis based on 200 core gene sequences allowed the classification of strains as PA14- or PAO1-like strains, illustrated in the color coding in orange (PA14) and blue (PAO1), PA7 and MHH13682 (shown in black) could not be assigned to either strain family. The branch length is proportional to the number of substitutions per site.

Two extracellular carbohydrate-binding proteins, LecA (PA-IL) and LecB (PA-IIL), are virulence factors and necessary for adhesion and biofilm formation.^8^–^10^ Disruption of the bacterial biofilm or inhibition of virulence factor function by blocking the lectins LecA or LecB (reviewed in ref. 11 and 12) is therefore an anti-virulence approach. LecA binds to d-galactose; LecB from the strain PAO1 shows high binding affinities for l-fucose (**1**) and reduced binding to d-mannose (**4**) and their glycoconjugates.^13^,^14^ Glycan-based LecB-directed inhibitors are able to disrupt established biofilms *in vitro*.^15^,^16^ To date, all reported LecB inhibitors focussed on the less virulent model strain *P. aeruginosa* PAO1 only (see for example ref. 15–22). The highly virulent *P. aeruginosa* patient isolate PA14 (ref. 23) has gained increasing attention recently. Despite a high genomic variability among the two strains and multiple other clinical isolates, the *lecA* and *lecB* genes are part of the commonly shared core genome.^24^,^25^ While the LecA sequence is conserved in these two model strains, the sequence of LecB shows a high degree of variation (Fig. 1A). Current anti-virulence drugs are directed towards LecB from PAO1, however, the efficacy of these therapies for other clinical isolates is a concern due to the observed variability of LecB, especially in the highly pathogenic strain PA14.

## Results and discussion

### Sequence analysis of clinical isolates: LecB as strain marker

The genes of both *P. aeruginosa* lectins *lecA* and *lecB* are part of the core genome^25^ and while LecA is highly conserved, LecB shows sequence variation in 15 amino acids in PA14 compared to PAO1 (Fig. 1A). To analyze the variability of LecB among patient isolates, we compared LecB sequences of our collection of clinical isolates.^26^ From these 151 isolates, a total of 79 strains showed a complete *lecB* transcriptomics sequence coverage. Five strains with mutations in the *lecB* gene resulting in frame shifts and thus in truncated amino acid sequences were discarded. The amino acid sequences of the remaining 74 strains were submitted to a multiple sequence alignment and the LecB sequences of *P. aeruginosa* strains PAO1, PA14, LESB58, PACS2 and PA7 obtained from Pseudomonas Genome Database^27^ were included in the analysis (Fig. S1†). The resulting phylogenetic tree (Fig. 1B), solely based on the LecB sequence, groups the strains into one branch containing the sequence of LecB_PAO1_ and a second branch containing LecB_PA14_, the sequence of the taxonomical outlier PA7 (ref. 28) is comparatively isolated. Surprisingly, the sequence distribution in this alignment of LecB sequences showed a striking correlation with an independent phylogenetic analysis^26^ of these clinical isolates based on 200 core genes, grouping the strains into two major families, PAO1-like and PA14-like (Fig. 1B, blue for PAO1 and orange for PA14). The sequence alignment shows that the sequence variations among the clinical isolates vary specifically at 15 positions where LecB_PA14_ and LecB_PAO1_ differ (Fig. S1,† see Fig. 1A for the individual mutations). In the taxonomical outlier PA7, sequence variations include two additional sites, not observed in any of the other strains. Besides those distinct differences observed between PA14 and PAO1, further variations are only present at two additional sites among all clinical isolates analyzed: A48G in 24 strains and A105S in 5 strains. Moreover, these additional substitutions are fully restricted to the PA14-like group. These findings show that the LecB sequence can serve as a genetic marker to classify clinical isolates into either PAO1- or PA14-like strains, a knowledge potentially useful for choosing a therapeutic action plan.

### Carbohydrate specificity

**Fig. 2:**
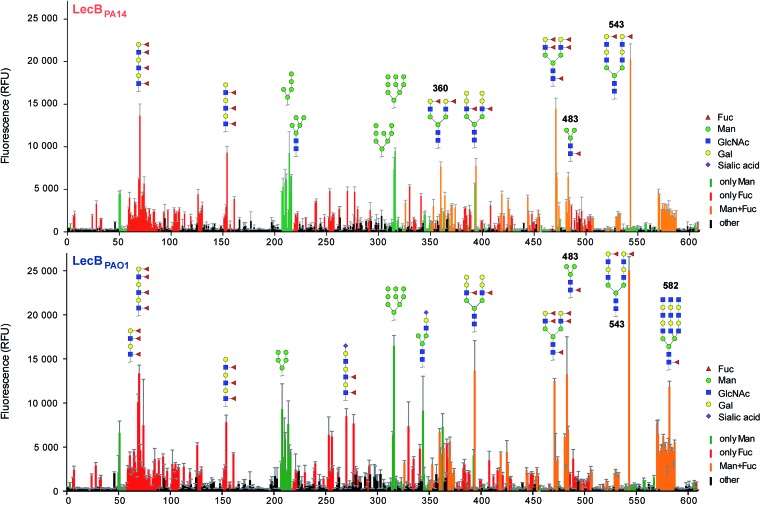
Profiling of glycan binding specificities of LecB_PA14_ (top) and LecB_PAO1_ (bottom) on the CFG mammalian glycan array. Glycans containing d-mannose are colored in green, those containing l-fucose in red and oligosaccharides with both moieties are colored in orange. Selected structures showing highest apparent affinities are illustrated in CFG notation.

Three amino acid variations are either in direct proximity to (S23A, G97S) or may influence local secondary structure (P73S) at the carbohydrate binding site and could directly impact ligand binding specificity and affinity. LecB_PA14_ was cloned, recombinantly expressed and purified from *E. coli*. The carbohydrate specificity of LecB_PA14_ and potential differences in selectivity compared to LecB_PAO1_ was analyzed on the CFG mammalian glycan array, which provided a spectrum of 609 different carbohydrate epitopes.^29^ Both FITC-labeled lectins were analyzed and, surprisingly, the profile of the glycan binding specificities was very similar for both lectins with only subtle differences detected (Fig. 2 and S2†). Both lectins showed comparable binding to fucosylated oligosaccharides, such as fucosylated *N*-glycans and fucosylated *N*-acetyl lactosamine repeats, as well as to high-mannose-type structures. LecB_PAO1_ showed stronger binding to core-fucosylated structures, such as glycans 483 and 582. Interestingly, the highest apparent affinity of both LecB variants was detected for a bi-antennary H type II antigen on a di-LacNAc *N*-linked glycan structure (no. 543, Fig. 2 and S3†). This high affinity could result from a simultaneous bivalent binding to two binding sites on the tetramer, which is supported by its shortened but otherwise identical LacNAc analog (no. 360) showing a reduced apparent affinity in this assay.

**Fig. 3:**
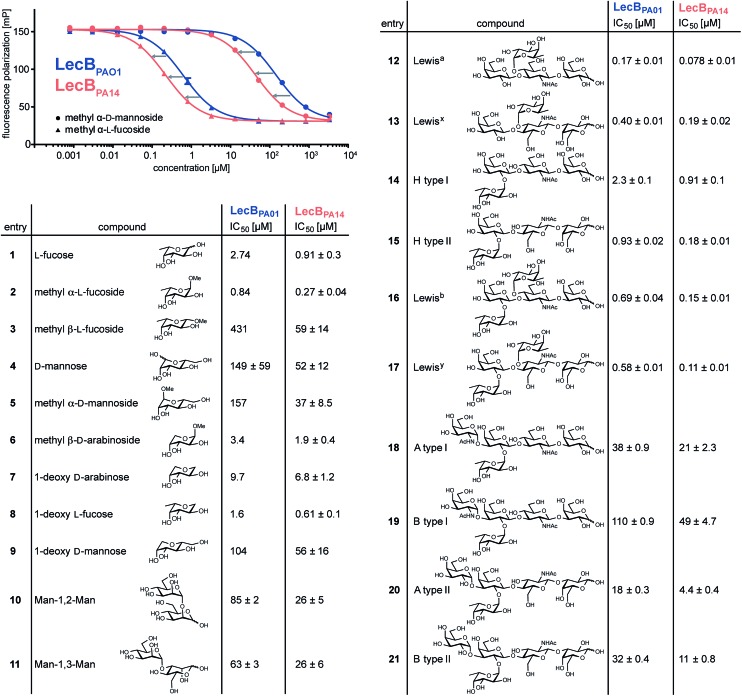
Biophysical evaluation of LecB_PA14_ and LecB_PAO1_ by a competitive binding assay. Data for LecB_PAO1_ with **1–3**, **5–9** are from the literature.^18^,^20^

To quantify the binding affinities of the carbohydrates we adapted the competitive binding assay for LecB_PA14_ (Fig. S5†) previously described for LecB_PAO1_.^18^ Because the glycan array showed very similar carbohydrate specificities for both lectins, 21 selected ligands previously shown to bind to LecB_PAO1_ were analyzed for their potential of inhibiting LecB_PA14_ (Fig. 3 and S5†). Generally, three- to seven-fold lower IC_50_ values were obtained for the interaction between LecB_PA14_ and all tested mono- or disaccharides (**1–11**) than observed for LecB_PAO1_. Previously, the trisaccharide Lewis^a^ (**12**), an antigen of the Lewis blood group system, was identified as a high affinity monovalent ligand for LecB_PAO1_ (*K*_d_ 212 nM).^30^ Furthermore, compound 543 (structure see Fig. S3†) identified from the glycan array screen bears a bi-antennary H type II blood group antigen, and therefore, the binding affinities of various blood group antigens were also examined (**12–21**, Fig. 3). The Lewis and H-type antigens (**12–17**) displayed nanomolar affinities. Lewis^a^ (**12**) demonstrated very high affinity as a monovalent lectin ligand (IC_50_ 78 nM) for LecB_PA14_ which was approximately two-fold higher than with LecB_PAO1_ (IC_50_ 166 nM). The addition of galactose (blood group A, **18**, **20**) or *N*-acetyl galactosamine (blood group B, **19**, **21**) to the H-type antigen, and fucose to Lewis^a^ or Lewis^x^ (resulting in Lewis^b^**16** and Lewis^y^**17**, respectively) led to reduced affinities.

**Fig. 4:**
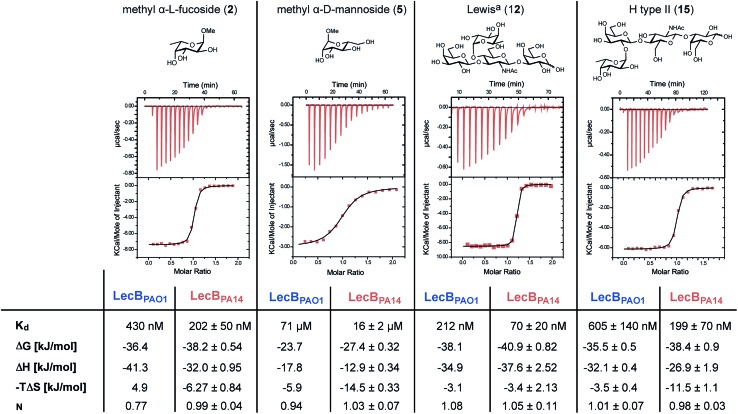
Isothermal titration calorimetry (ITC) of LecB_PA14_ with **2**, **5**, **12** and **15** and comparison with the titration of **15** against LecB_PAO1_ or literature data for **2**, **5** and **12** with LecB_PAO1_.^14^,^30^

The interactions of methyl α-l-fucoside (**2**), methyl α-d-mannoside (**5**), the high affinity ligand Lewis^a^ (**12**) and the H-type II antigen (**15**) with LecB_PA14_ were further studied by isothermal microcalorimetry (Fig. 4, S6 and S7†). The interaction of **15** with LecB_PAO1_ was also analyzed by ITC (Fig. 4 and S7†). Generally, increased affinities of the ligands to LecB_PA14_ were observed by two- to three-fold lower *K*_d_ values for LecB_PA14_ compared to LecB_PAO1_. The high affinity of the Lewis^a^ antigen (**12**, IC_50_ 78 nM) observed in the competitive binding assay was confirmed (*K*_d_ 70 nM for LecB_PA14_) and resulted from mainly enthalpy driven binding. To the best of our knowledge, this is the highest affinity observed for native monovalent glycan ligands and lectins in general. Stronger lectin binding affinities of monovalent ligands have been reported only for lipophilic glycoconjugates with FimH^31^,^32^ or cholera toxin,^33^ or glycomimetics.^34^ In contrast to LecB_PAO1_, the binding of methyl α-l-fucoside (**2**, *K*_d_ 202 nM for LecB_PA14_), methyl α-d-mannoside (**5**, *K*_d_ 16 μM for LecB_PA14_) and the H-type II antigen (**15**, *K*_d_ 199 nM for LecB_PA14_) revealed reduced enthalpic contributions to binding compensated with favorable entropic contribution for LecB_PA14_.

### Synthesis and evaluation of a bivalent *N*-glycan ligand for LecB

**Scheme 1:**
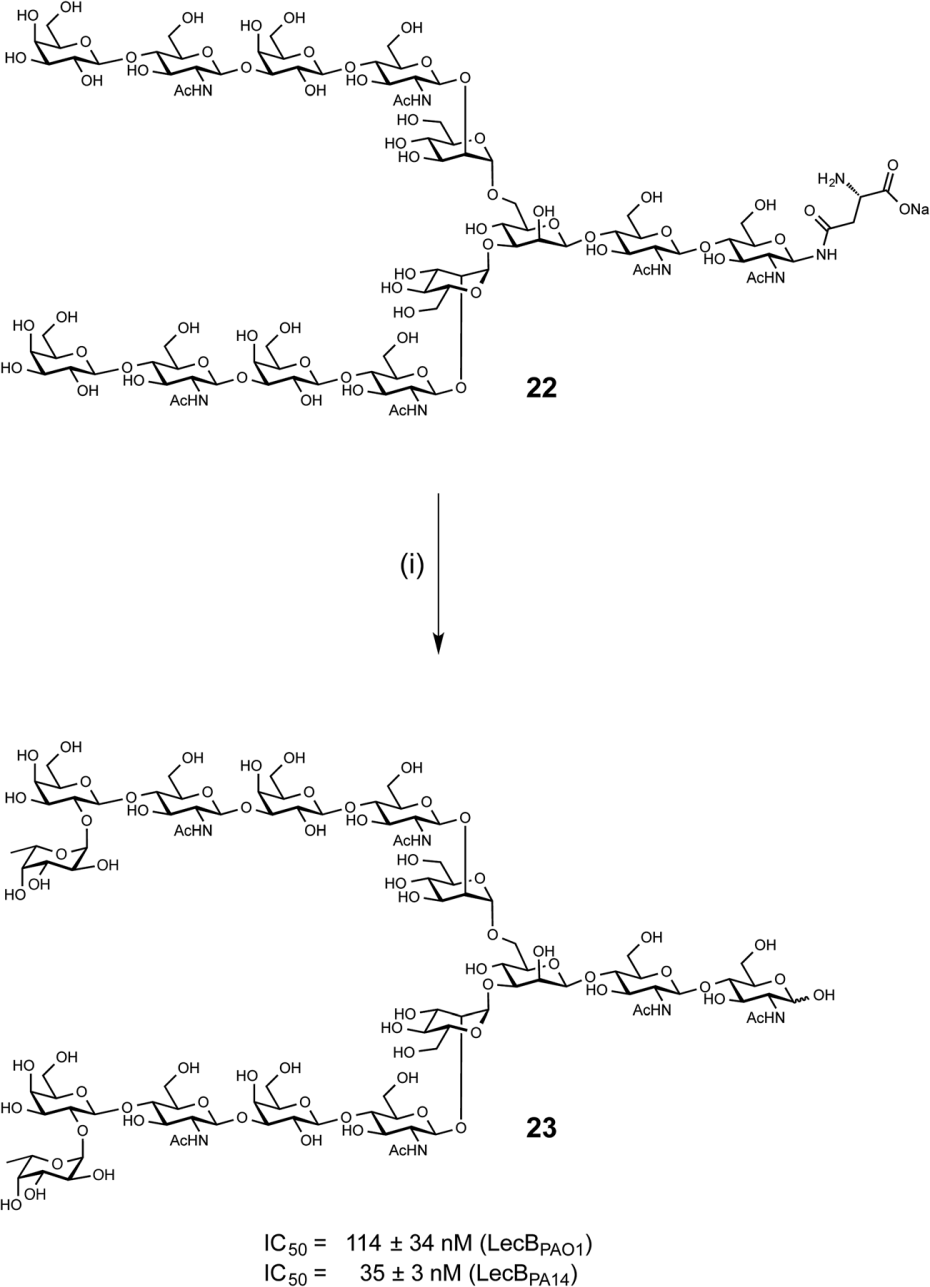
Chemoenzymatic synthesis of bivalent ligand **23** and inhibition of LecB_PAO1_ and LecB_PA14_. (i) GDP-Fuc, FUT-II (culture medium), Tris buffer, 37 °C, 72 h.

LecB from both PAO1 and PA14 showed strong binding on the glycan microarray to compound 543 which is a biantennary di-LacNAc *N*-linked glycan bearing the H-type II antigen on the terminus of both branches (see **23**, Scheme 1). Glycosylated asparagine **22** (ref. 35) bearing the biantennary di-LacNAc *N*-linked glycan in presence of GDP-fucose was exposed to the insect cell culture supernatant of a baculovirus-infected expression culture of FUT-II^36^ (Scheme 1). The reaction required extended reaction times (72 h) for completion, leading to a removal of the asparagine moiety through other components in the complex culture media. However, sufficient quantities of the difucosylated free oligosaccharide **23** were obtained as pure compound in 20% yield after two chromatographic purifications. **23** was then tested in the competitive binding assays. Compound **23** was found to be a potent ligand for both LecB_PAO1_ (IC_50_ = 114 nM) and LecB_PA14_ (IC_50_ = 35 nM) (Scheme 1), with a strong avidity for LecB compared to its monovalent derivative **15**, displaying IC_50_s of 930 and 180 nM, respectively.

**Fig. 5:**
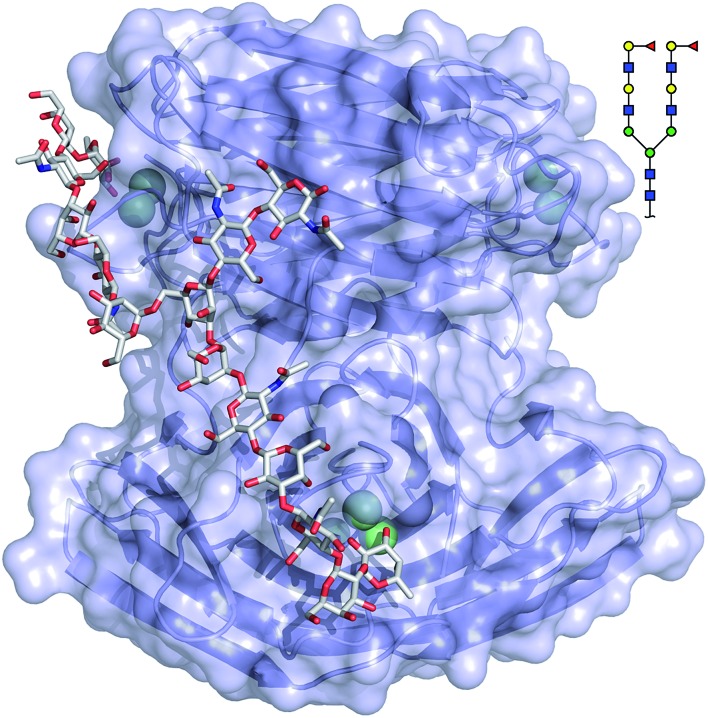
Docking of the bi-antennary H type II ligand (CFG compound 543) to LecB_PAO1_. This bivalent ligand can simultaneously bind to two binding sites of LecB_PAO1_, which are presented on the vertices of a tetrahedron in the tetramer. Carbohydrate ligands and amino acid side chains are shown as sticks colored by elements (C: grey, N: blue, O: red). Ca^2+^-ions are shown as green spheres.

The binding mode of compound 543 of the mammalian glycan array was analyzed by molecular docking experiments with LecB_PAO1_. Docking of the bi-antennary H type II ligand **23** to LecB_PAO1_ revealed that a bivalent binding mode is possible (Fig. 5). Here, both fucosides in one ligand molecule can simultaneously bind to two binding sites on the vertices of one LecB tetramer. The arrangement of the two extended di-LacNAc antenna of this *N*-glycan allows optimal positioning of the two fucose residues into two very distant binding sites of the tetramer. In contrast to reports for other multimeric lectins, *e.g.* wheat germ agglutinin,^37^ shiga toxin,^38^ LecA^39^,^40^ and others, this is the first proposal of a chelating binding mode for LecB.

### The crystal structure of LecB from *P. aeruginosa* PA14

**Fig. 6:**
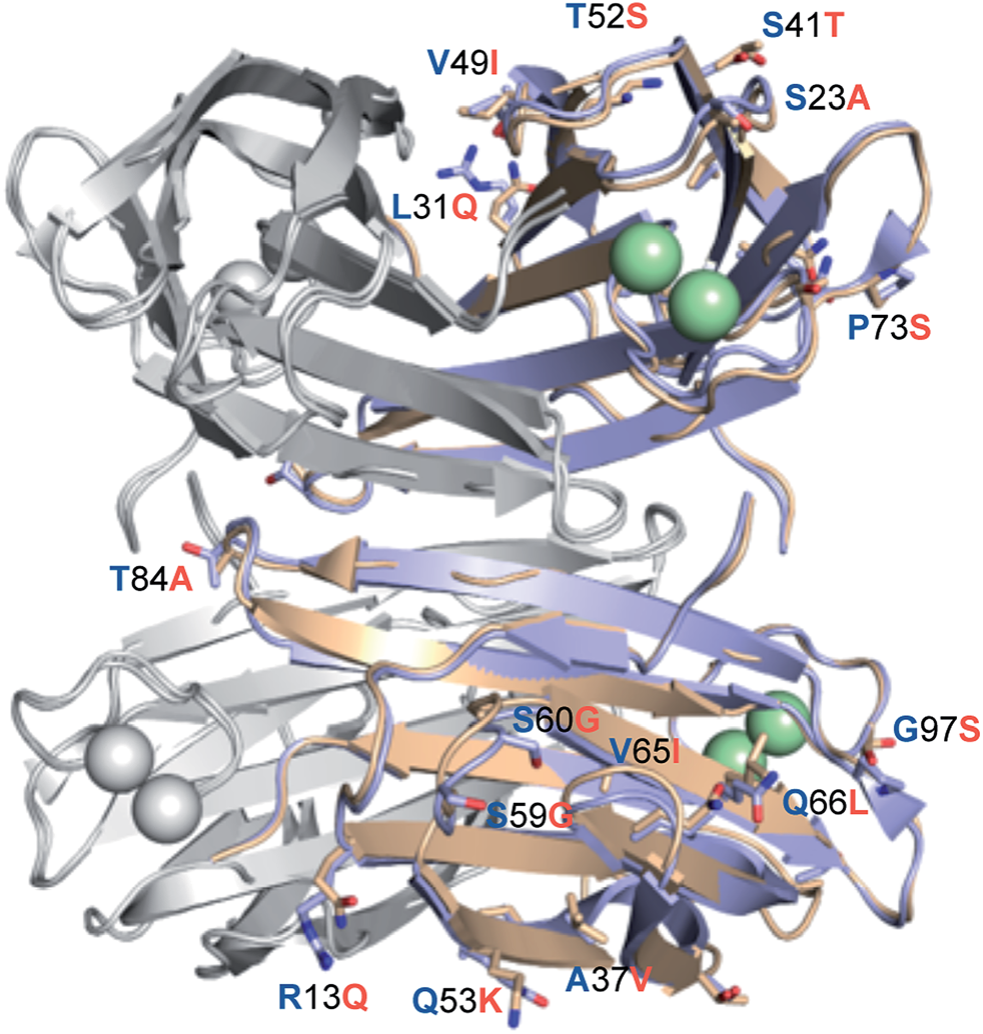
Crystal structure of LecB_PA14_. Superposition of the crystal structures LecB_PA14_ (orange, resolution 1.70 Å) and LecB_PAO1_ (blue; PDB code: ; 1OXC 44). For clarity of illustration, differing amino acids are not shown in each monomer but are depicted only once per tetramer as labeled sticks (amino acid one letter code: blue for PAO1, orange for PA14) and colored by elements (N: blue, O: red). Ca^2+^-ions are shown as green spheres.

LecB_PA14_ was subsequently crystallized and its structure was solved by X-ray crystallography (Fig. 6 and Table S1†). In analogy to LecB_PAO1_,^41^ the 1.70 Å resolution native structure of LecB_PA14_ shows the lectin as homotetramer with the four carbohydrate recognition domains (CRD) containing two Ca^2+^-ions each located on the vertices of a pseudotetrahedron (Fig. 6). A direct binding of G114 to one Ca^2+^-ion from the neighboring monomer stabilizes the tetrameric structure of the lectin, which was confirmed in solution by dynamic light scattering experiments (Fig. S4†). Notably, the structural alignment of LecB_PA14_ and LecB_PAO1_ revealed that all amino acid variations in LecB_PA14_ are located exclusively on the outer surface of the tetramer (Fig. 6). Mutations at the interfaces of the tetramer subunits are not present, and thus the oligomerization of LecB is conserved. Oligomerization of lectins is a prerequisite for their function as cross-linking agents, a feature that is probably important for the biological function of LecB.^17^,^42^

### Structure of LecB_PA14_ in complex with carbohydrate ligands

**Fig. 7:**
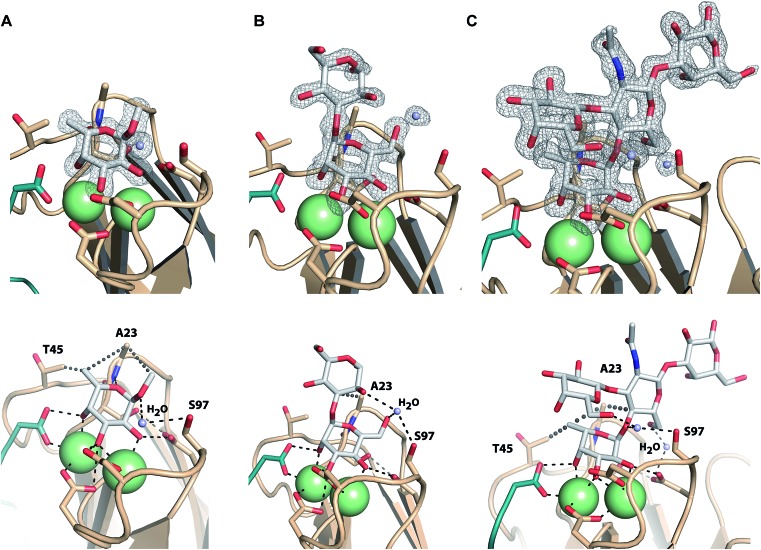
Crystal structures of LecB_PA14_ in complex with **2** (A), **11** (B) and **12** (C). (Upper panel) Display of electron density for the ligands at 1σ. Ca^2+^-ions are shown as green spheres. ((A) lower panel) Methyl α-l-fucoside (**2**) establishes a lipophilic interaction with T45 and A23. The crystal structure reveals a water-mediated hydrogen bond between the anomeric oxygen and S97 and an additional hydrophobic interaction of the aglycon with A23. ((B) lower panel) The crystal structure with α-1,3-d-mannosyl d-mannose (**11**) indicates one water mediated hydrogen bond between the 6-OH group and S97, as well as an interaction of the same water with the 2-OH group of the second mannose. An additional lipophilic contact between the aglycon and A23 is observed. ((C) lower panel) The tetrasaccharide Lewis^a^ (**12**) establishes water-mediated hydrogen bonds *via* two water molecules and lipophilic contacts with T45 and A23. The C-terminus of each chain involved in the CRD of the neighboring chain is shown as cartoon in green. Ca^2+^-ions are shown as green spheres.

Co-crystals of LecB_PA14_ were obtained and the structures of LecB_PA14_ in complex with methyl α-l-fucoside (**2**, Fig. 7A), α-1,3-d-mannosyl d-mannose (**11**, Fig. 7B), and the high affinity ligand Lewis^a^ (**12**, Fig. 7C) were solved by X-ray crystallography at resolutions between 1.40 and 1.55 Å (Table S1†). Both Ca^2+^-ions in LecB_PA14_ mediate the binding with the carbohydrates by complexation of three hydroxy groups in the saccharide ligand. Additionally, a direct contact of these hydroxy groups with D96, D99 and the C-terminal G114 of the neighboring monomer is observed. In the fucose-containing ligands (**2**, **12**) the C-6 methyl group is embedded in a lipophilic pocket formed by T45 and A23. This latter variation in the CRD, S23A, allows additional van der Waals interactions between A23 and aglycones of the Ca^2+^-coordinated carbohydrate moiety. In contrast to LecB_PAO1_, the G97S variation in LecB_PA14_ offers an additional hydroxy group with hydrogen bonding potential in the CRD. In each of the three LecB_PA14_ complexes, the carbohydrate ligands establish one water-mediated hydrogen bond with S97. In addition to the interactions described, the high affinity ligand Lewis^a^ (**12**) forms numerous hydrogen bonds with LecB_PA14_, involving two water molecules that simultaneously establish several hydrogen bonds with protein and ligand. While the reducing end galactose of the tetrasaccharide Lewis^a^ does not interact with the protein, all other three monosaccharides in Lewis^a^ contribute to the binding to LecB_PA14_. One water-mediated interaction of GlcNAc O-6 with D96, as well as another water-mediated hydrogen bond between O-6 of the terminal d-galactose moiety in Lewis^a^ (**12**) with S97 are observed in the crystal structure. This latter water molecule additionally donates one hydrogen to establish a hydrogen bond with the anomeric oxygen of fucose. The extensive set of hydrophobic and hydrophilic interactions of Lewis^a^ with LecB_PA14_ forms the molecular basis for its high affinity binding.

Both sequence variations that are located directly in the carbohydrate binding site, S23A and G97S, are involved in extensive interactions with all carbohydrate ligands in LecB_PA14_ which is not observed in LecB_PAO1_ to this extent. Therefore, both variations are likely to contribute to the increased affinity of LecB_PA14_ for its ligands compared to LecB_PAO1_.

## Conclusion

The adhesins LecA and LecB are virulence factors of *P. aeruginosa* and play an important role in biofilm formation and thus chronic infections. To date, only LecB from PAO1 has been studied.^13^,^41^,^43^,^44^ The sequence of LecB, however, differs significantly between the evolutionarily diverged PAO1- or PA14-like strains. Here, we showed that its sequence is a direct marker and correlates with the classification of *P. aeruginosa* into either PA14- or PAO1-like family of strains. This property could serve clinically for rapid choice of the appropriate therapies in accordance to the strain family which have different virulence properties. Despite high sequence variations, biophysical characterization uncovered that both variants possess very similar glycan binding preferences, although very surprisingly LecB_PA14_ generally displays 2- to 7-fold higher affinities. LecB_PA14_ showed a very high affinity of 70 nM for Lewis^a^, an unprecedented high affinity for a monovalent natural glycan–lectin interaction in general. Chemoenzymatic synthesis of a common high affinity ligand identified in the glycan array screen allowed evaluation of **23** in the competitive binding assays and potencies up to 35 nM for LecB_PA14_ were observed. Due to the high avidity of **23** and a docking model with LecB, this is first bivalent ligand likely to bridge simultaneously two binding sites in the tetramer of LecB. The crystal structure of LecB_PA14_ and comparison to its PAO1 homolog revealed the localization of all mutated amino acids on the outer surface of the LecB tetramer some of which are located in or near the carbohydrate binding site. The molecular basis for the superior affinity of LecB_PA14_ for its carbohydrate ligands was established on the basis of three crystal structures of LecB_PA14_ in complex with its ligands, revealing an extensive set of attractive interactions that are possible due to the two amino acid variations in the carbohydrate binding site.

Importantly, based on the knowledge obtained in this study, carbohydrate-derived inhibitors targeting LecB of both *P. aeruginosa* families, PAO1 and PA14, that equally constitute the collection of strains analyzed, can now be developed into future anti-adhesion therapeutics against a broad range of clinical isolates.

The evolutionary reasons for the diverged protein sequence families (LecB from PAO1 *vs.* PA14) remain unclear. One hypothesis is that the LecB_PA14_ compensates for the absence of the exopolysaccharide psl in PA14. However, analysis of the clinical isolates showed that only PA14 lacks *pslA-D* from the *psl* operon, whereas other strains in this family generally possessed the full psl operon.^26^ Whether the highly conserved sequence variations between the PAO1- and PA14-like LecB sequence families and the resulting higher affinities for carbohydrate ligands are directly responsible for the enhanced virulence of *P. aeruginosa* PA14 compared to PAO1, as observed for the pathoadaptive adhesin FimH in *E. coli*,^45^ is subject to future investigations.

## Materials and methods

### Chemicals

d-Mannose (**4**) and methyl α-d-mannoside (**5**) were purchased from Sigma Aldrich (Germany), l-fucose (**1**), α-1,2-d-mannosyl d-mannose (**10**), α-1,3-d-mannosyl d-mannose (**11**) from Dextra Laboratories (Reading, UK), methyl β-d-arabinoside (**6**) from TCI Europe, methyl α-l-fucoside (**2**) and methyl β-l-fucoside (**3**) from Carbosynth Ltd. (UK). Blood group antigens **12–21** were purchased from Elicityl OligoTech (France).

### Chemoenzymatic synthesis of bivalent ligand α-l-fucopyranosyl-(1→2)-β-d-galactopyranosyl-(1→4)-2-acetamido-2-deoxy-β-d-glucopyranosyl-(1→3)-β-d-galactopyranosyl-(1→4)-2-acetamido-2-deoxy-β-d-glucopyranosyl-(1→2)-α-d-mannopyranosyl-(1→3)-(α-l-fucopyranosyl-(1→2)-β-d-galactopyranosyl-(1→4)-2-acetamido-2-deoxy-β-d-glucopyranosyl-(1→3)-β-d-galactopyranosyl-(1→4)-2-acetamido-2-deoxy-β-d-glucopyranosyl-(1→2)-α-d-mannopyranosyl-(1→6))-β-d-mannopyranosyl-(1→4)-2-acetamido-2-deoxy-β-d-glucopyranosyl-(1→4)-2-acetamido-2-deoxy-d-glucopyranose (**23**)

Compound **22** (ref. 35) (11.9 mg, 4.76 μmol) and GDP-Fuc (11.2 mg, 19.1 μmol, 4 eq.) were dissolved in 500 μL Tris–HCl buffer (100 mM, pH 7.5, 20 mM MgCl_2_). FUT-II^36^ culture media (∼10 U mmol^–1^ substrate) was added then the reaction was incubated for 48 hours at 37 °C. Additional GDP-Fuc (2 eq.) and FUT-II were added and the reaction continued for 24 hours. The reaction was centrifuged and the supernatant subjected to size exclusion chromatography using a Sephadex™ G-25 column (1 × 140 cm), equilibrated and eluted with H_2_O. Fractions containing product were combined and lyophilized. The solid was dissolved in H_2_O then subjected to further purification using an All-Tech™ Carbograph column (150 mg) eluted with H_2_O with increasing concentration of methanol (0 to 50%) to give pure α/β fucosylated product **23** (2.5 mg, 20%) as a white amorphous solid. The asparagine aglycone of **22** was hydrolyzed during the reaction; *R*_f_ 0.05 (6 : 3 : 2, iPrOH–H_2_O–NH_4_OH); ^1^H NMR (D_2_O, 600 MHz) *δ* 5.22 (2H, m), 5.11 (1H, s, H-1 GlcNAc-α-OH), 5.04 (1H, s), 4.85 (1H, s), 4.63–4.61 (2H, m), 4.54–4.46 (5H, m), 4.38 (2H, m), 4.17–4.03 (6H, m), 3.91–3.39 (81H, m), 2.00 (3H, s), 1.96 (15H, m), 1.15 (6H, m); MS (MALDI-TOF) *m*/*z* [M + Na]^+^ calcd for C_102_H_170_N_6_NaO_74_ 2686, obs 2688.

### Sequence analysis

The amino acid sequences of the *P. aeruginosa* strains (clinical isolates^26^ and reference strains^27^) were submitted to a multiple sequence alignment resulting in a phylogenetic tree using the ; http://phylogeny.lirmm.fr platform.^46^ The alignment was performed using MUSCLE,^47^ Gblocks for alignment curation^48^ and PhyML for phylogeny.^49^ See Fig. S1, ESI† for the alignment.

### Bacterial strains and growth conditions

*E. coli* XL1 blue was used for amplification and *E. coli* BL21 (DE3) for expression of plasmid pRS01.4 carrying the sequence of LecB_PA14_. Bacteria were grown in lysogeny broth (LB) supplemented with ampicillin (100 mg L^–1^).

### Molecular cloning and expression of LecB_PA14_

Genomic DNA from *P. aeruginosa* UCBPP-PA14 was isolated using Gen Elute Bacterial Genomic DNA Kit (Sigma Aldrich). PCR amplification was performed with Phusion polymerase (New England Biolabs) and primers introducing NdeI (5′-GGAATTCCATATGGCAACAAGGAGTG-3′) and HindIII (5′-CCCAAGCTTCTAGCCGAGCGGCCAG-3′) restrictions sites. After digestion with NdeI and HindIII restriction enzymes (New England Biolabs) the DNA fragment was ligated into the multiple cloning site of digested pET22b(+) (Novagen) with T4 DNA ligase (New England Biolabs) and resulted in plasmid pRS01.4. The sequence was confirmed by sequencing (GATC Biotech) with primers T7 promotor (5′-TAATACGACTCACTATATAGG-3′) and T7 terminator (5′-GCTAGTTATTGCTCAGCGG-3′). Expression and purification of the protein was performed in analogy to LecB_PAO1_ (ref. 18) and the protein was dialyzed against TBS/Ca (20 mM Tris, 137 mM NaCl, 2.6 mM KCl at pH 7.4 supplemented with 1 mM CaCl_2_) and stored at –20 °C.

### Fluorescence labeling of LecB and CFG mammalian glycan array

Each LecB variant (700 μL, 58 μM in Na_2_CO_3_ buffer, pH 9.3) was incubated at r.t. under shaking (500 rpm) with FITC (33 μL, 3 mg mL^–1^, in Na_2_CO_3_ buffer, pH 9.3) for 1 h. Purification was performed as described for unlabeled protein above, the protein concentration was determined as described in Fig. S2.† FITC-labeled LecB_PAO1_ and LecB_PA14_ were tested on the Consortium for Functional Glycomics (CFG) mammalian glycan array (Core H) version 5.2. Standard procedures of Core H were run at 200 μg mL^–1^, 20 μg mL^–1^, and 2 μg mL^–1^ protein concentration.

### Dynamic light scattering (DLS) measurements

DLS measurements were performed on a Zetasizer Nano-ZS (Malvern Instruments, UK). Stock solutions were filtered with a syringe filter before measurements. 50 μL of LecB (100 μM) in TBS/Ca (20 mM Tris, 137 mM NaCl, 2.6 mM KCl at pH 7.4 supplemented with 1 mM CaCl_2_) was measured at 25 °C.

### Competitive binding assay

The competitive binding assay based on fluorescence polarization was performed as described previously for PAO1.^18^ Briefly, 20 μL of a stock solution of LecB_PA14_ (150 nM) and fluorescent reporter ligand *N*-(fluorescein-5-yl)-*N*′-(α-l-fucopyranosyl ethylen)-thiocarbamide (15 nM) in TBS/Ca (20 mM Tris, 137 mM NaCl, 2.6 mM KCl at pH 7.4 supplemented with 1 mM CaCl_2_) were mixed with 10 μL serial dilutions (1 mM to 12.8 nM) of testing compounds in TBS/Ca in triplicates in black 384-well microtiter plates (Greiner Bio-One, Germany, cat no 781900). After addition of the reagents, the plate was incubated for 8–22 h at r.t. in a humidity chamber. Fluorescence emission parallel and perpendicular to the excitation plane was measured on a PheraStar FS (BMG Labtech, Germany) plate reader with excitation filters at 485 nm and emission filters at 535 nm. The measured intensities were reduced by buffer values and fluorescence polarization was calculated. The data were analyzed using BMG Labtech MARS software and/or with Graphpad Prism and fitted according to the four parameter variable slope model. Bottom and top plateaus were defined by the standard compounds l-fucose (**1**) and methyl α-d-mannoside (**5**) respectively and the data was reanalyzed with these values fixed. A minimum of three independent measurements of triplicates each was performed for every ligand.

### Isothermal titration calorimetry (ITC)

The concentration of the monomer of LecB_PA14_ (dissolved in TBS/Ca (20 mM Tris, 137 mM NaCl, 2.6 mM KCl at pH 7.3 supplemented with 1 mM CaCl_2_)) was determined by UV spectroscopy at 280 nm using a molar extinction coefficient of 6990 M^–1^ cm^–1^ (obtained from ProtParam^50^). The temperature of the sample cell was 25 °C. The titration was performed with a solution of ligands in the same buffer, concentrations of protein and ligands is given in Fig. S6 and S7.† ITC was performed on a Microcal ITC200 (GE) and the data was analyzed according to the one site binding model using the Microcal Origin software. A minimum of three independent titrations was performed for each ligand.

### Docking

Bi-antennary oligosaccharide 543 was built in the Sybyl graphic editor (Certara) using the monosaccharides structures available on the glyco3D portal (http://glyco3D.cermav.cnrs.fr). One fucose was located in the binding site as observed in crystal structure of LecB_PAO1_ (ref. 41) and each monosaccharide was stepwise included with conformation in agreement with published energy maps^51^ and general direction towards one of the two neighboring binding site. Only one of the two sites was accessible after building the whole oligosaccharide in extended conformation. Final optimization was performed with distance constraints to maintain both fucoses in the crystallographic position and energy minimization with the Tripos force-field.

### Crystallization and structure determination

For crystallization of LecB_PA14_ with ligands **2**, **11** or **12**, a protein solution in water (10 mg mL^–1^) was incubated in a 9 : 1 ratio with compound (10 mM compound in 20 mM Tris, 137 mM NaCl, 2.6 mM KCl at pH 7.3 supplemented with 1 mM CaCl_2_) for minimum 1 h prior crystallization. Crystallization was performed by the hanging drop vapor diffusion method using 1 μL of protein plus ligand + 1 μL of reservoir solution at 19 °C in a 24 well plate. Crystals were formed after one or two days and were frozen in liquid nitrogen after the addition of appropriate cryoprotectant (Table S1†). Data were collected on beamline BM30A at ESRF (Grenoble) using a ADSC Q315r CCD detector apart from the structure of **11**, which was collected on ID23-2 using a pilatus detector. Data were processed using XDS and all further computing was performed using the CCP4 suite.^52^,^53^ All structures were solved by molecular replacement using PHASER.^54^ The tetramer coordinates of PDB ; 1UZV were used as model for the native structure of LecB_PA14_ which was then used as model for all the complex structures. Manual corrections of the model were performed using Coot^55^ and interspersed with cycles of maximum likelihood refinement with REFMAC5.8.^56^ Coordinates and structure factors have been deposited in the Protein Data Bank under accession codes ; 5A6Q, ; 5A6X, ; 5A6Y, ; 5A6Z.

## Author contributions

R. S. and S. W. cloned and generated recombinant protein; R. S. and A. V. performed the structural-biology experiments; R. S. performed binding assays; A. I. performed modeling experiments; S. W. analyzed glycan array data and sequencing data; C. N. and J. P. synthesized divalent H-type II antigen, A. K. and S. H. provided sequencing data; A. I. and S. H. gave conceptual advice; R. S., S. W. and A. T. conceived the experiments and wrote the paper.

## Conflict of interest

The authors declare no competing financial interest.

## Supplementary Material

Supplementary information
