# Supplementary material for: The virulence factor LecB varies in clinical isolates: consequences for ligand binding and drug discovery
Source: Chem Sci. 2016 May 11;7(8):4990–5001. doi: 10.1039/c6sc00696e (PMC6018602; doi:10.1039/c6sc00696e)
Supplement: Supplementary file 1 [file SC-007-C6SC00696E-s001.pdf]

Electronic Supplementary Material (ESI) for Chemical Science.
This journal is © The Royal Society of Chemistry 2016

Supporting Information to

The virulence factor LecB varies in clinical isolates: consequences for ligand binding and drug discovery

Roman Sommer[1,2#], Stefanie Wagner[1,2#], Annabelle Varrot[3], Corwin M. Nycholat[4], James C. Paulson[4], Ariane Khaledi[5], Susanne Häussler[5], Anne Imberty[3], Alexander Titz[1,2*]

[1] Chemical Biology of Carbohydrates, Helmholtz Institute for Pharmaceutical Research Saarland (HIPS), D-66123 Saarbrücken, Germany

[2] Deutsches Zentrum für Infektionsforschung (DZIF), Standort Hannover–Braunschweig (Germany)

[3] Centre de Recherche sur les Macromolécules Végétales (CERMAV-UPR5301), CNRS and Université Grenoble Alpes, BP53, F-38041 Grenoble cedex 9, France

[4] Department of Cell and Molecular Biology and Department of Chemical Physiology, The Scripps Research Institute, 10550 North Torrey Pines Road, La Jolla, CA 92037, USA

[5] Molecular Bacteriology, Helmholtz Centre for Infection Research, D-38124 Braunschweig, Germany

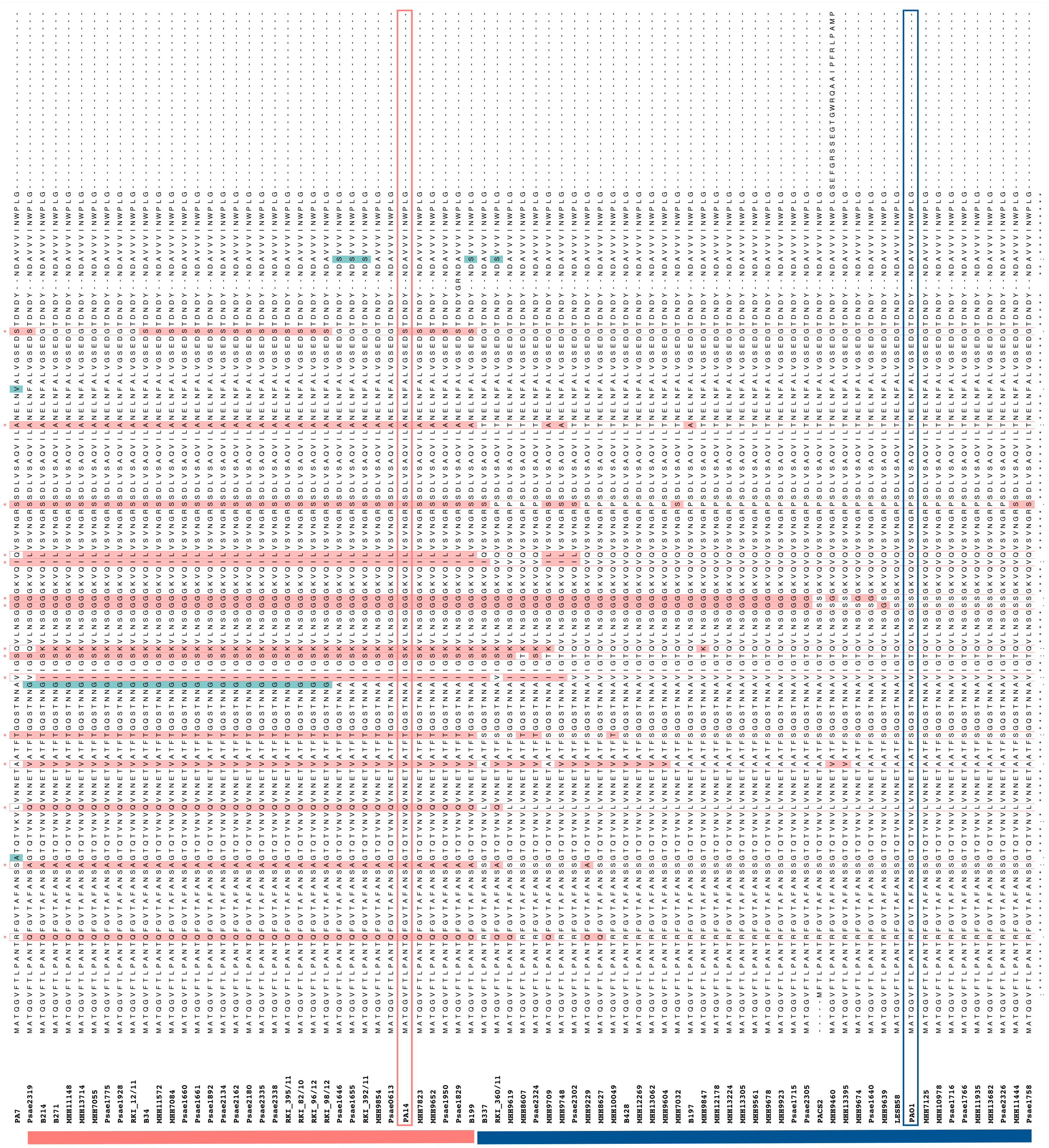

Figure S1: Multiple sequence alignment (MUSCLE) of LecB sequences and color coded PA14-like strains (red) and PAO1-like strains (blue). The point mutations present in the sequence of $LecB_{PA14}$ compared to $LecB_{PAO1}$ are highlighted with asterisks (red). Additional point mutations that are not present in PA14 are highlighted in green. Amino acid numbering corresponds to the crystal structure of $LecB_{PAO1}$, which lacks methionine.

UV straight calibration line

A280

µg/mL	A280	A280	A280	M A280	STD A280	µM
30	1,690	1,646	1,528	1,621	0,084	77,046
15	0,919	1,004	0,884	0,936	0,062	38,523
7,5	0,473	0,525	0,446	0,481	0,040	19,261
3,75	0,245	0,247	0,213	0,235	0,019	9,631
1,87	0,166	0,117	0,084	0,122	0,041	4,803
0,93	0,049	0,091	0,038	0,059	0,028	2,388
0,47	0,020	0,021	0,025	0,022	0,003	1,207

A495

µg/mL	A495	A495	A495	M A495	STD A495	rA495/A280
30	1,375	1,495	1,280	1,383	0,108	0,853
15	1,523	1,270	1,538	1,444	0,151	1,543
7,5	1,108	1,105	0,977	1,063	0,075	2,209
3,75	0,599	0,598	0,520	0,572	0,045	2,435
1,87	0,317	0,320	0,238	0,292	0,047	2,384
0,93	0,149	0,172	0,095	0,139	0,040	2,337
0,47	0,068	0,071	0,048	0,062	0,013	2,833

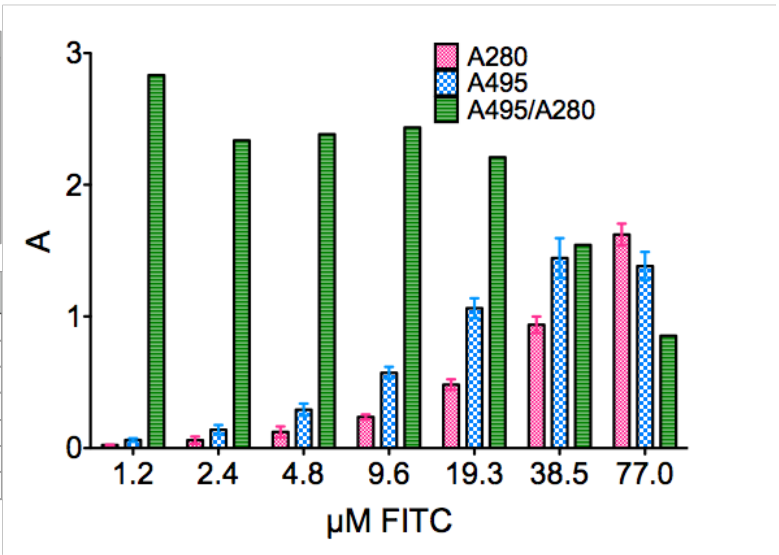

LINEAR RANGE: 0-20 µM FITC in TBS/Ca

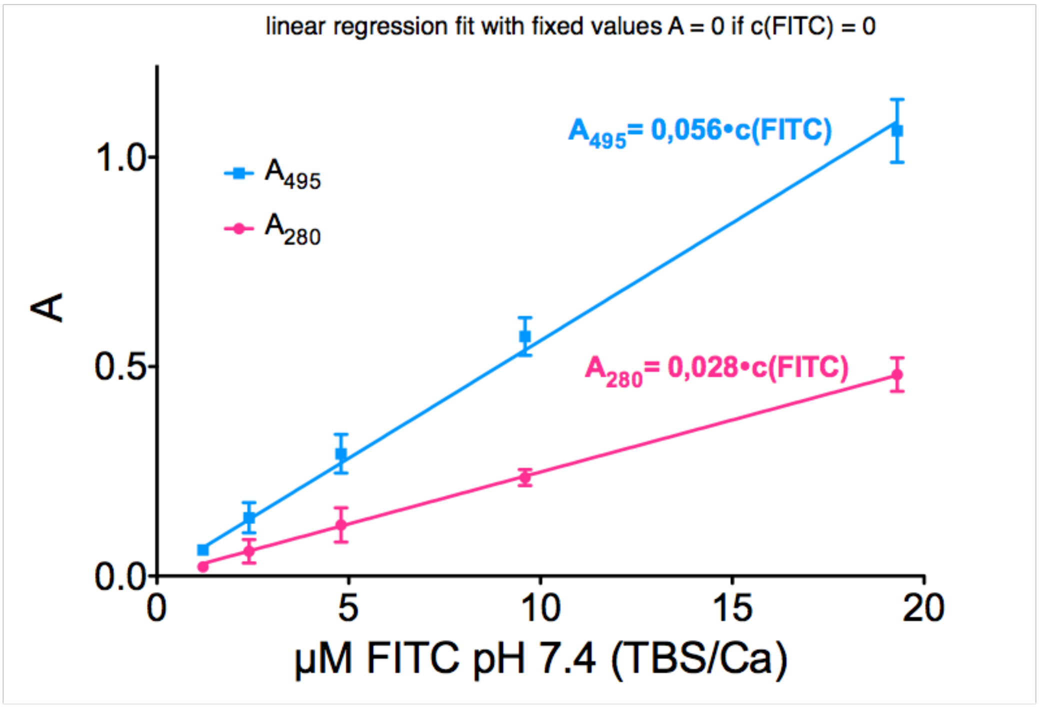

$$c_{labeled\ LecB} = \frac{A_{280} - 0.028 \cdot \frac{A_{495}}{0.056}}{6990}$$

Figure S2: concentration determination of FITC-labeled lectin $LecB_{PAO1}$ and $LecB_{PA14}$ by UV absorbance measurements

bi-antennary H type II blood group antigen **543**
from mamalian glycan array

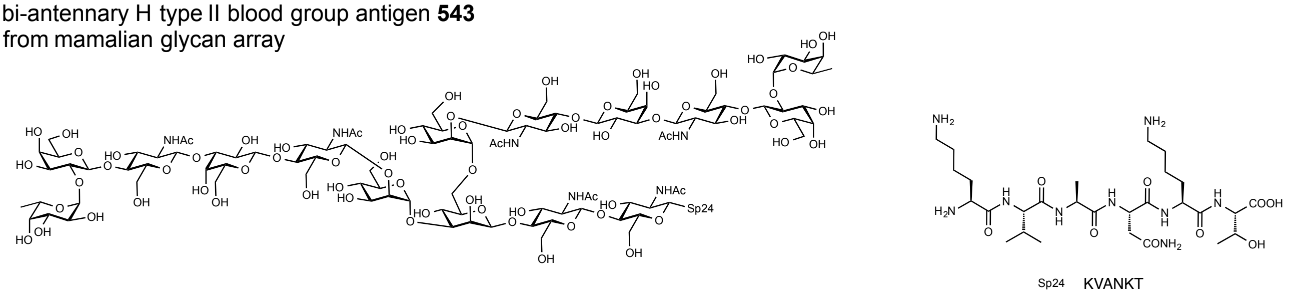

Figure S3: Structure of the best ligand identified by glycan array screening (compound 543).

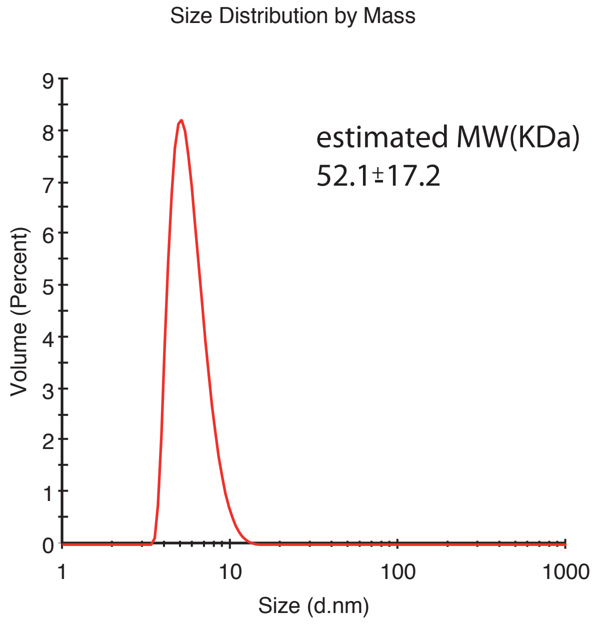

Figure S4: Dynamic light scattering of LecB$_{PA14}$ suggested a tetramer in solution.

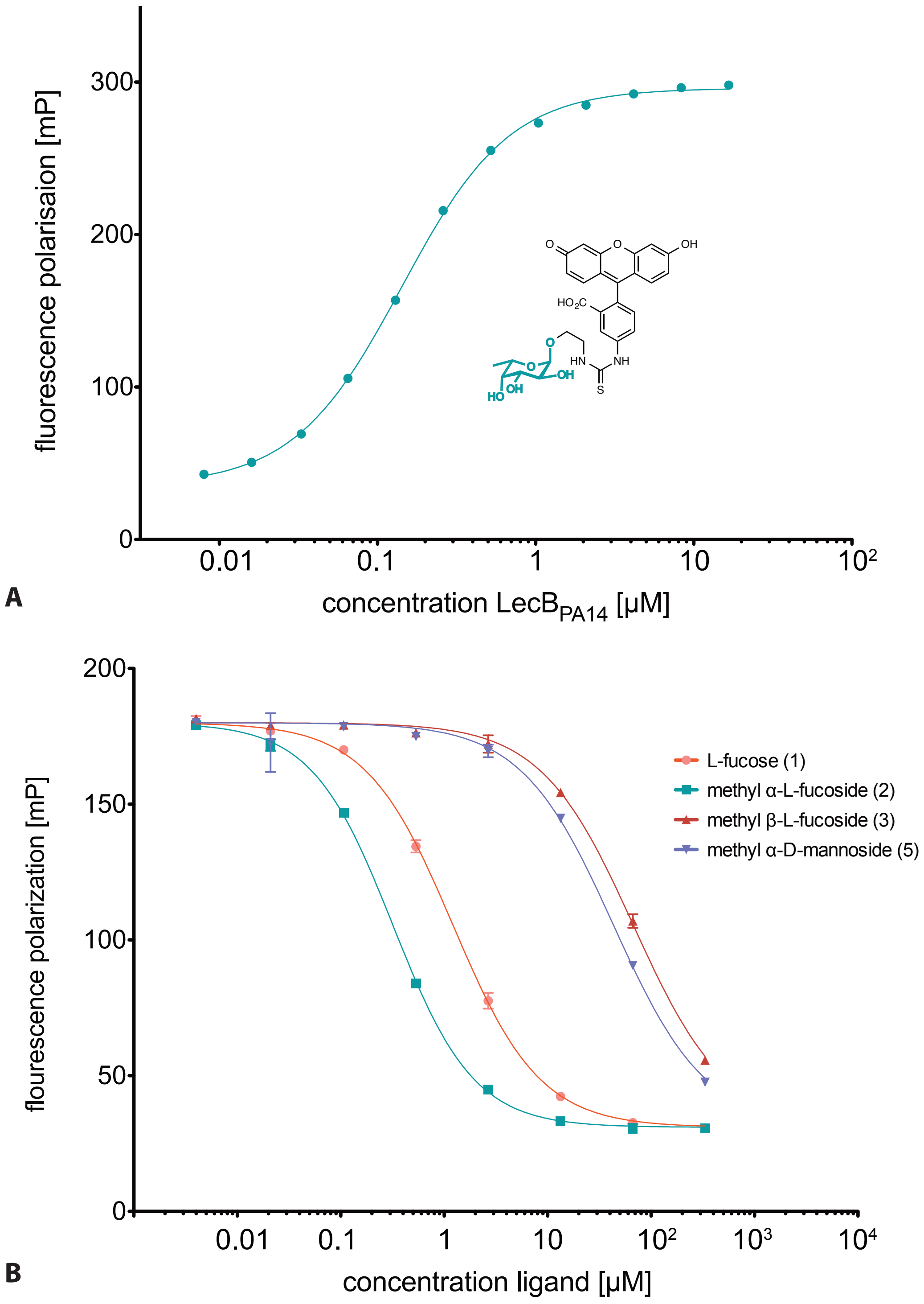

Figure S5: (A) Titration of fluorescein-labeled fucose-based reporter ligand N-(fluorescein-5-yl)-N'-(α-L-fucopyranosyl ethylen)-thiocarbamide (Hauck *et al*, ACS Chem. Biol., 2013) at 10 nM with $LecB_{PA14}$ and determination of the fluorescence polarization. (B) Competitive inhibition of the binding of N-(fluorescein-5-yl)-N'-(α-L-fucopyranosyl ethylen)-thiocarbamide (10 nM) to $LecB_{PA14}$ (100 nM) with various ligands. One representative titration of triplicates is shown and error bars shown here represent standard deviation of triplicates on one plate.

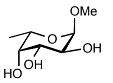 **methyl α-L-fucoside (2)**	$LecB_{PA14}$ 91 µM<br>methyl α-L-fucoside (**2**) 800 µM<br>TBS supplemented with 1 mM $CaCl_2$	$LecB_{PA14}$ 91 µM<br>methyl α-L-fucoside (**2**) 800 µM<br>TBS supplemented with 1 mM $CaCl_2$	$LecB_{PA14}$ 236 µM<br>methyl α-L-fucoside (**2**) 2.3 mM<br>TBS supplemented with 1 mM $CaCl_2$	$LecB_{PA14}$ 110 µM<br>methyl α-L-fucoside (**2**) 1 mM<br>TBS supplemented with 1 mM $CaCl_2$
	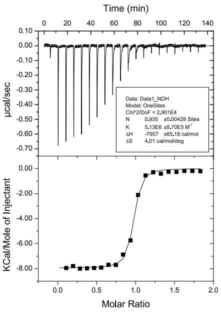	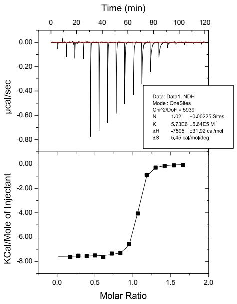	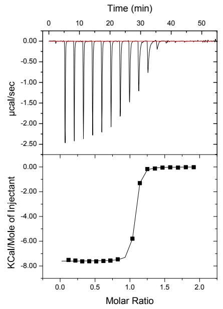	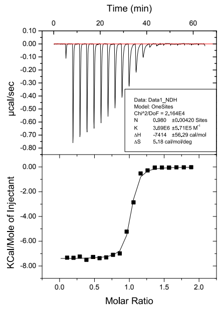
**Ka [1/M]**	5,13E+06	5,73E+06	5,94E+06	3,69E+06
**△H [cal/mol]**	-7957	-7593	-7612	-7414
**△s [cal/molK]**	4,01	5,45	5,47	5,18
**Kd [M]**	1,95E-07	1,75E-07	1,68E-07	2,71E-07
**N**	0,935	1,02	1,03	0,980
**△G [cal/mol]**	-9,15E+03	-9,22E+03	-9,24E+03	-8,96E+03

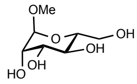 **methyl α-D-mannoside (5)**	$LecB_{PA14}$ 236 µM<br>methyl α-D-mannoside (**5**) 2.3 mM<br>TBS supplemented with 1 mM $CaCl_2$	$LecB_{PA14}$ 236 µM<br>methyl α-D-mannoside (**5**) 2.3 mM<br>TBS supplemented with 1 mM $CaCl_2$	$LecB_{PA14}$ 499 µM<br>methyl α-D-mannoside (**5**) 5.0 mM<br>TBS supplemented with 1 mM $CaCl_2$
	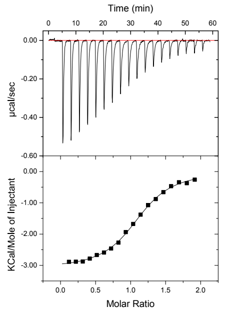	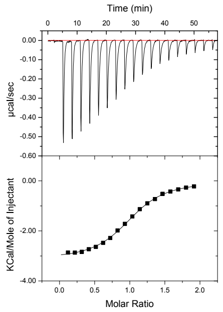	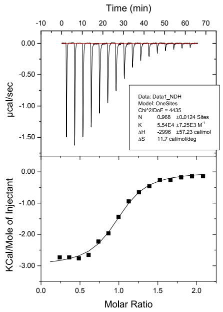
**Ka [1/M]**	7,18E+04	6,20E+04	5,54E+04
**△H [cal/mol]**	-3112	-3153	-2996
**△s [cal/molK]**	11,8	11,3	11,7
**Kd [M]**	1,39E-05	1,61E-05	1,81E-05
**N**	1,11	1,02	0,968
**△G [cal/mol]**	-6,63E+03	-6,52E+03	-6,48E+03

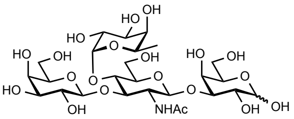 **Lewis^a**	$LecB_{PA14}$ 84.1 µM<br>Lewis^a (**12**) 800 µM<br>TBS supplemented with 1 mM $CaCl_2$	$LecB_{PA14}$ 94 µM<br>Lewis^a (**12**) 650 µM<br>TBS supplemented with 1 mM $CaCl_2$	$LecB_{PA14}$ 124 µM<br>Lewis^a (**12**) 1.1 mM<br>TBS supplemented with 1 mM $CaCl_2$
	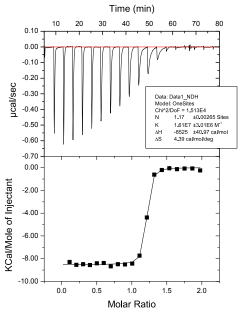	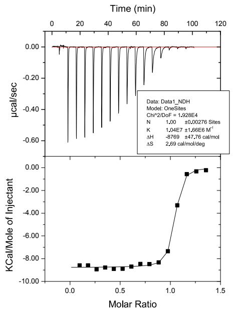	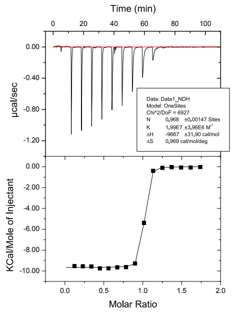
**Ka [1/M]**	1,61E+07	1,04E+07	1,99E+07
**△H [cal/mol]**	-8521	-8769	-9667
**△s [cal/molK]**	4,39	2,69	0,969
**Kd [M]**	6,21E-08	9,62E-08	5,03E-08
**N**	1,17	1,00	0,968
**△G [cal/mol]**	-9,83E+03	-9,57E+03	-9,96E+03

Figure S6: Isothermal microcalorimetry of $LecB_{PA14}$ with methyl α-L-fucoside (**2**), methyl α-D-mannoside (**5**) and with Lewis^a (**12**)

	LecB$_{PA14}$ 94 µM	LecB$_{PA14}$ 94 µM	LecB$_{PA14}$ 124 µM	LecB$_{PA14}$ 124 µM
	H-type II (**15**) 1 µM	H-type II (**15**) 1 µM	H-type II (**15**) 1 µM	H-type II (**15**) 1.1 µM
	TBS supplemented with 1 mM $CaCl_2$	TBS supplemented with 1 mM $CaCl_2$	TBS supplemented with 1 mM $CaCl_2$	TBS supplemented with 1 mM $CaCl_2$
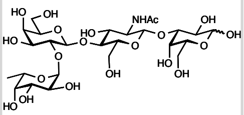 **H type II**	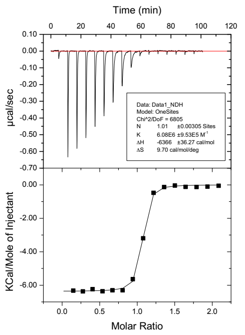	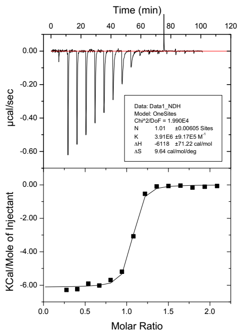	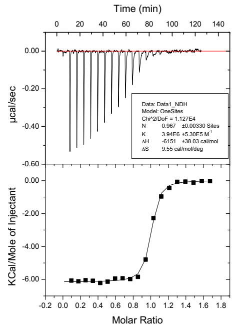	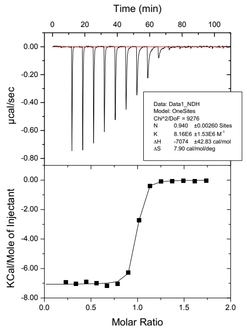
**Ka [1/M]**	6.08E+06	3.91E+06	3.94E+06	8.16E+06
**△H [cal/mol]**	-6366	-6118	-6151	-7074
**△s [cal/molK]**	9.7	9.6	9.6	7.9
**Kd [M]**	1.64E-07	2.56E-07	2.54E-07	1.23E-07
**N**	1.01	1.01	0.97	0.94
**△G [cal/mol]**	-9258	-8992	-8998	-9429

	LecB$_{PAO1}$ 139 µM	LecB$_{PAO1}$ 132 µM	LecB$_{PAO1}$ 124 µM
	H-type II (**15**) 1 µM	H-type II (**15**) 1 µM	H-type II (**15**) 1.3 µM
	TBS supplemented with 1 mM $CaCl_2$	TBS supplemented with 1 mM $CaCl_2$	TBS supplemented with 1 mM $CaCl_2$
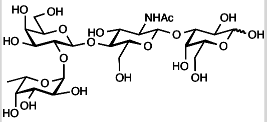 **H type II**	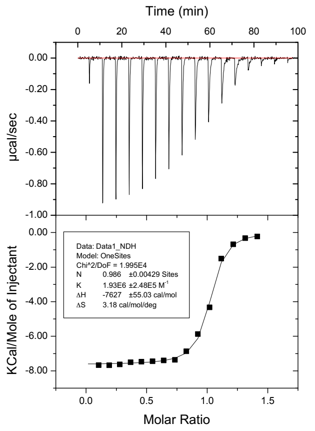	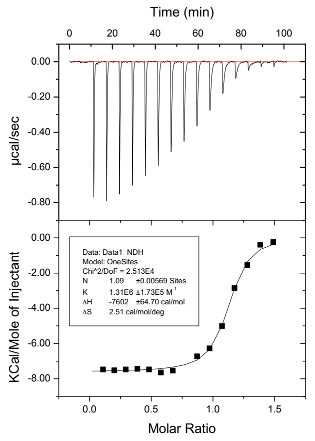	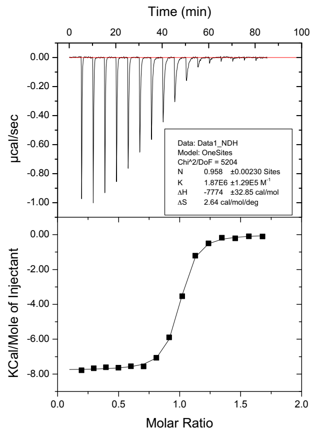
**Ka [1/M]**	1.93E+06	1.31E+06	1.87E+06
**△H [cal/mol]**	-7627	-7602	-7774
**△s [cal/molK]**	3.2	2.5	2.6
**Kd [M]**	5.18E-07	7.63E-07	5.35E-07
**N**	0.99	1.09	0.96
**△G [cal/mol]**	-8575	-8350	-8561

Figure S7: Isothermal microcalorimetry of LecB$_{PA14}$ (top row) and LecB$_{PAO1}$ (bottom row) with H-type II antigen (**15**).

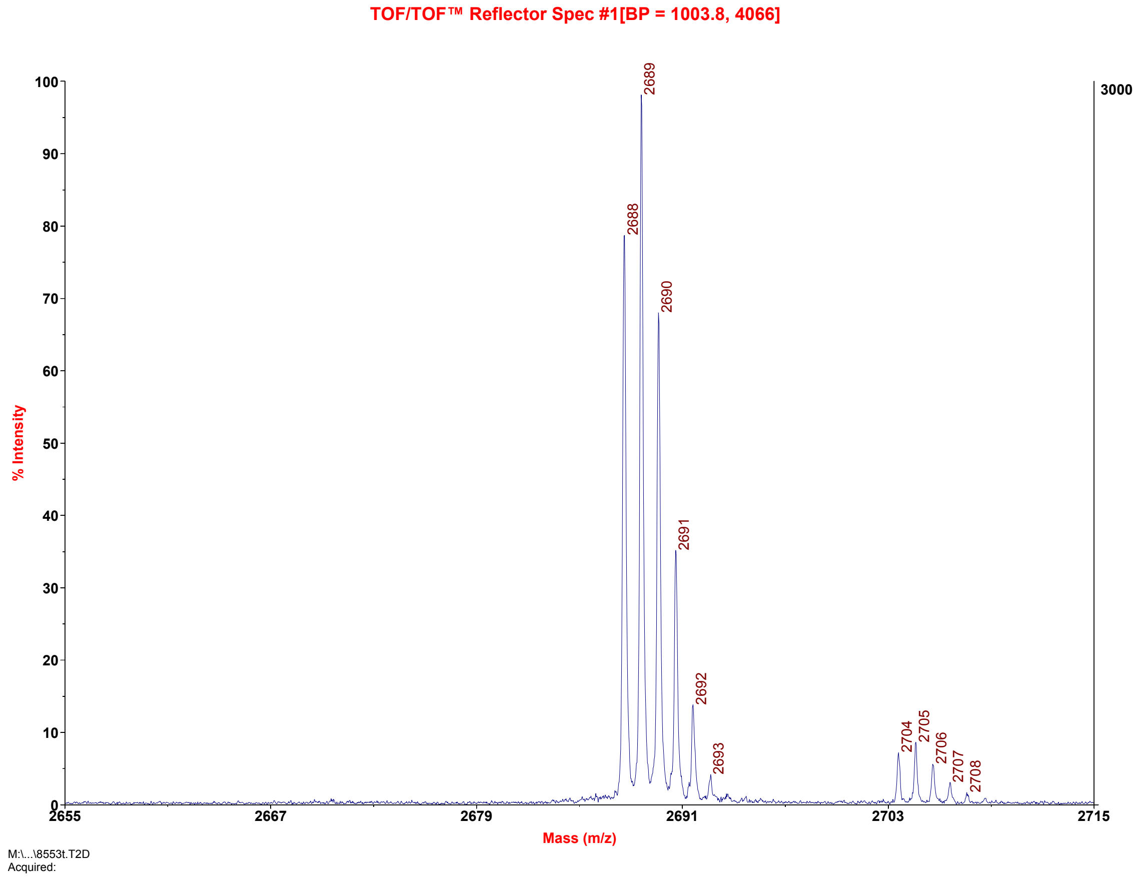

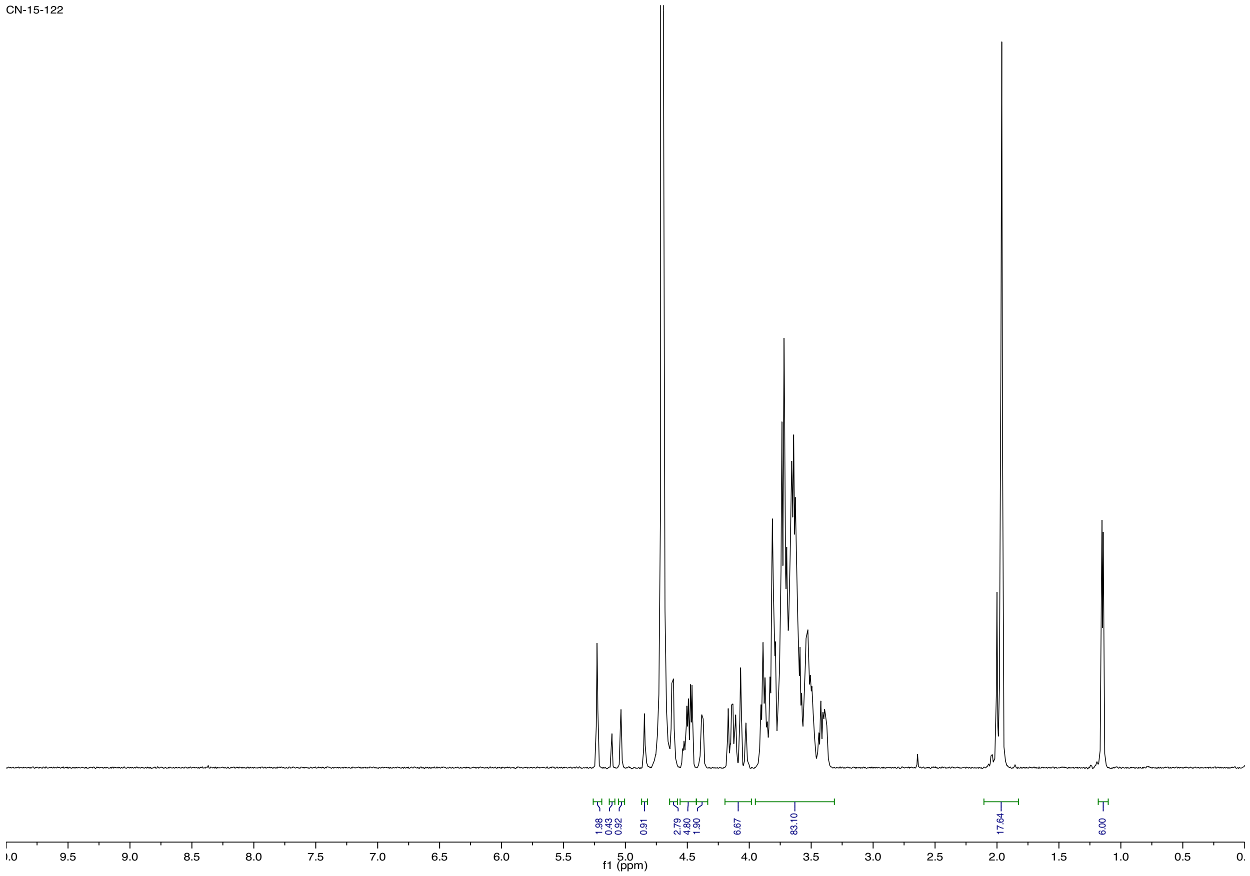

Figure S8: MALDI-MS (top) and 1H-NMR (bottom) of divalent N-glycan **23**.

Generic Display Report

Analysis Info

Analysis Name	D:\Data\CBCH\Roman\RS-b-303\RS-b-303MS_CN543.d	Acquisition Date	12/15/2015 4:43:46 PM
Method	MS-NglycanRS-b-303.m	Operator	cbch
Sample Name	RS-b-303AutoMSN-CN543	Instrument	amaZon SL
Comment			

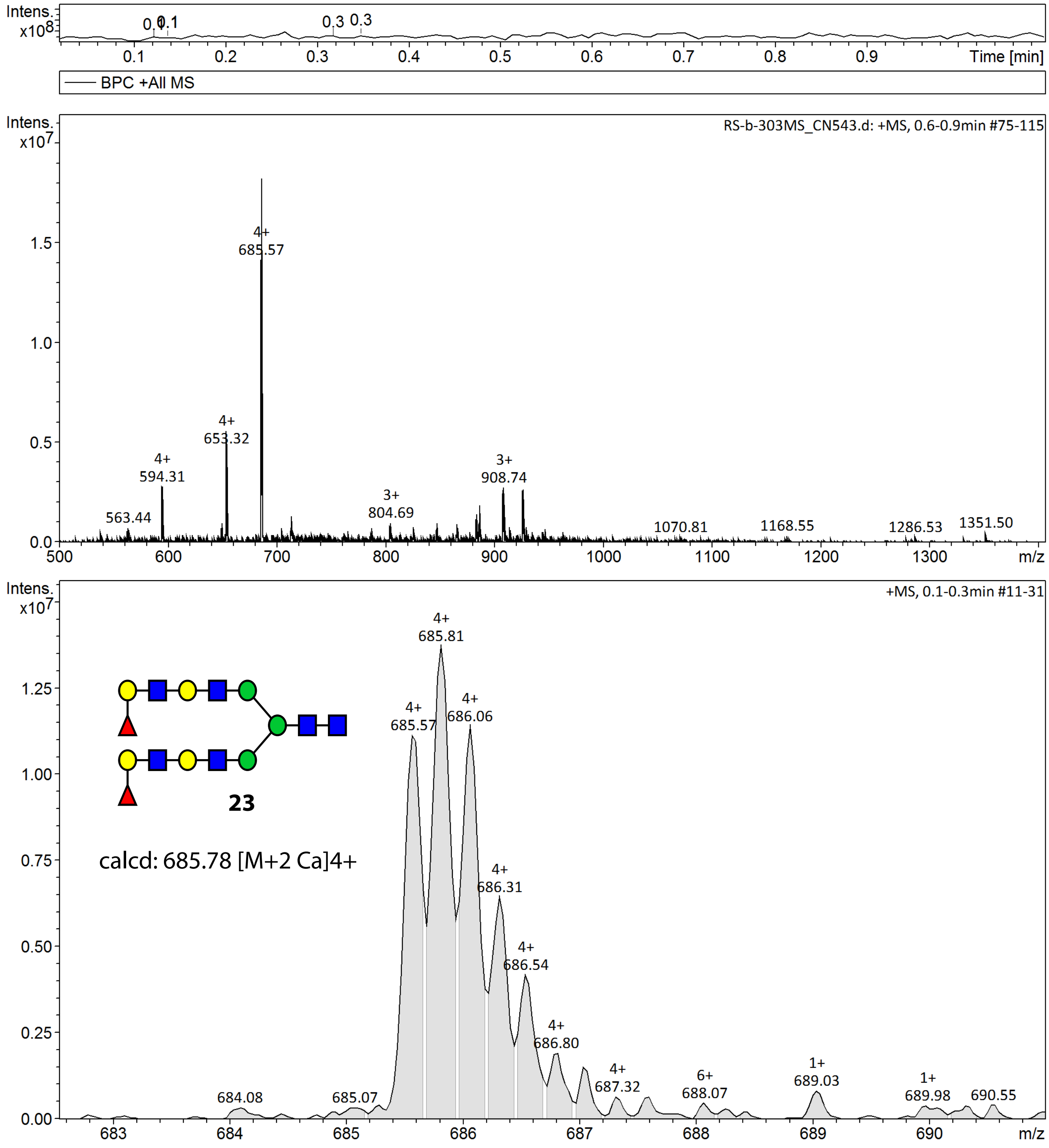

Figure S9: ESI-MS of of divalent N-glycan **23** in presence of 300 μM $CaCl_2$.

Generic Display Report

Analysis Info

Analysis Name	D:\Data\CBCH\Roman\RS-b-303\RS-b-303MSn-1351_CN543-3.d	Acquisition Date	12/15/2015 5:06:33 PM
Method	MSn-1351-NglycanRS-b-303.m	Operator	cbch
Sample Name	RS-b-303AutoMSN-CN543	Instrument	amaZon SL
Comment			

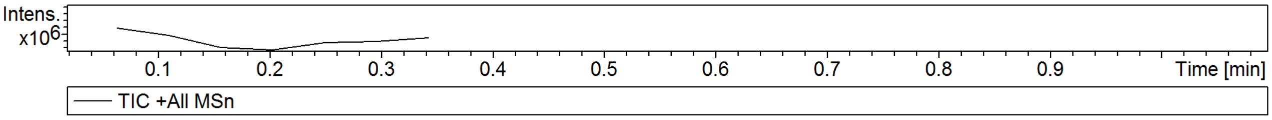

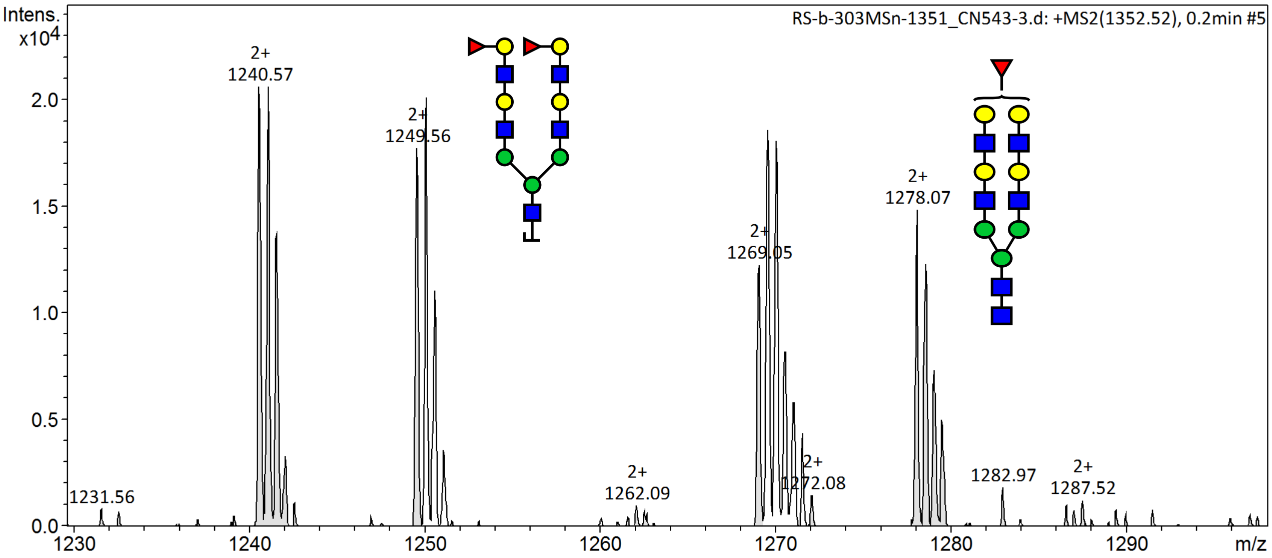

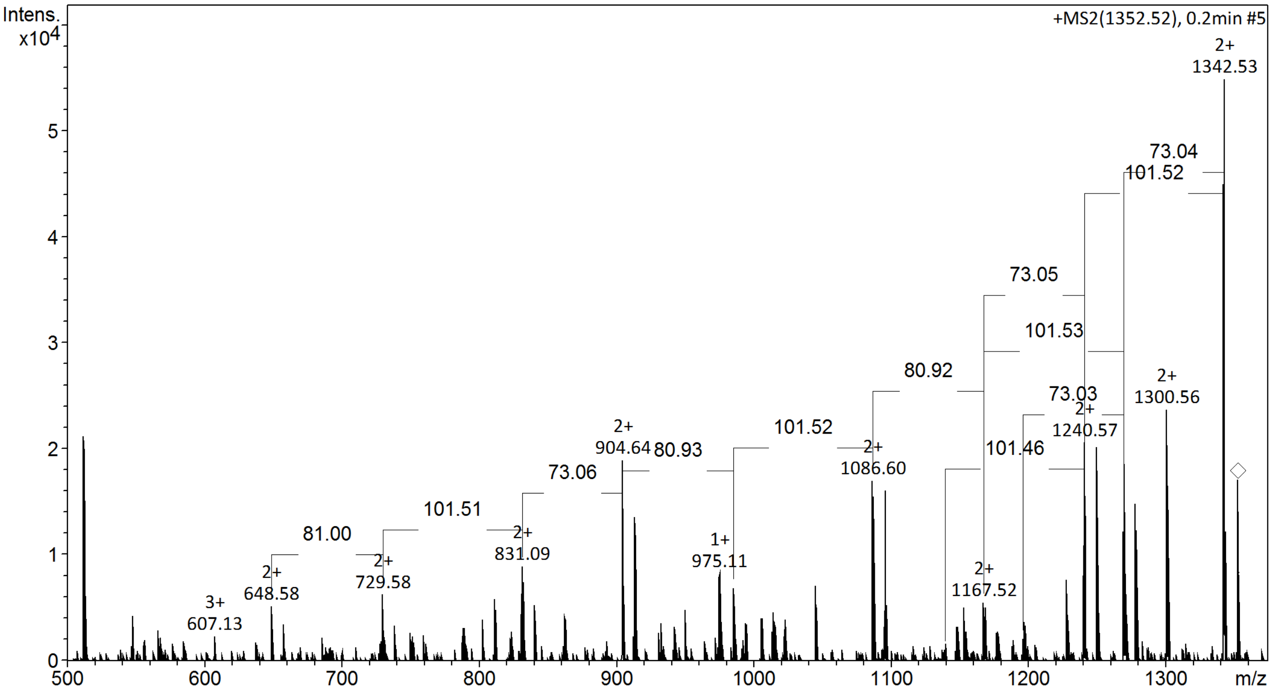

Figure S10: ESI-MS^2 of divalent N-glycan **23** in presence of 300 μM $CaCl_2$. m/z 1342.5 corresponds to $[M-H_2O+Ca]^{2+}$, loss of Fuc (**Δ**m/z = 73), Hex (**Δ**m/z = 81) and GlcNAc (**Δ**m/z = 101.5).

Generic Display Report

Analysis Info

Analysis Name	D:\Data\CBCH\Roman\RS-b-303\RS-b-303MSn-1351_CN543-3.d	Acquisition Date	12/15/2015 5:06:33 PM
Method	MSn-1351-NglycanRS-b-303.m	Operator	cbch
Sample Name	RS-b-303AutoMSN-CN543	Instrument	amaZon SL
Comment			

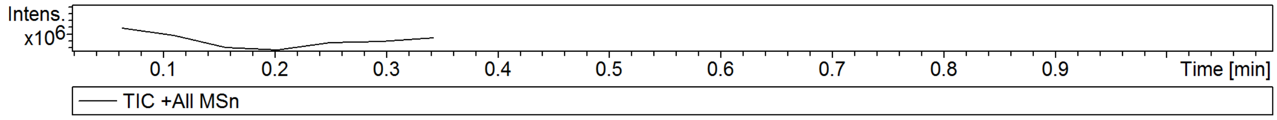

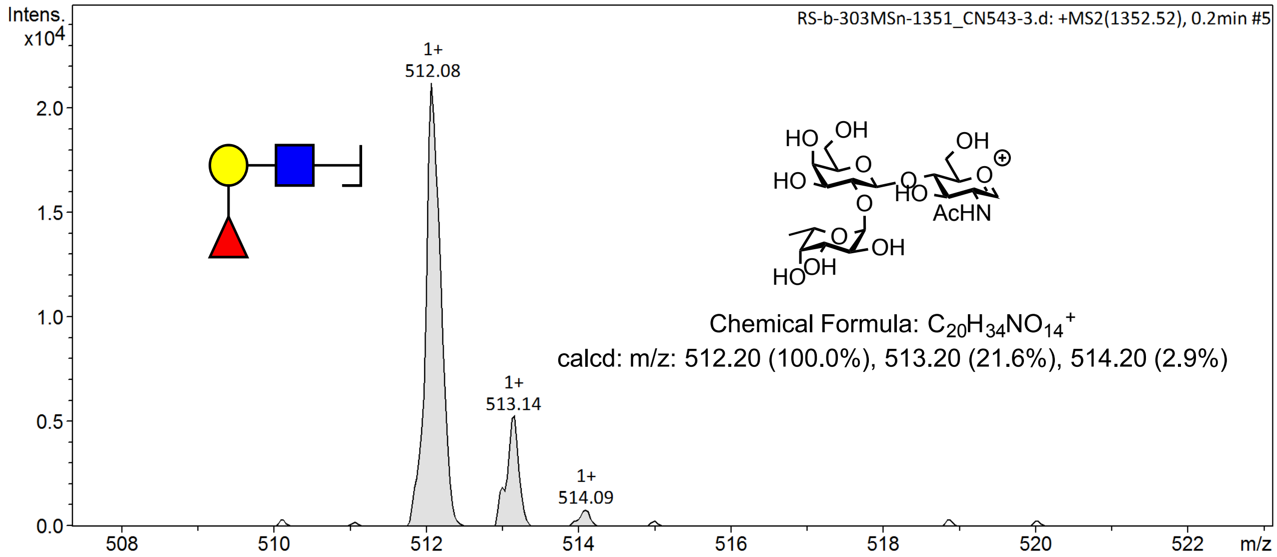

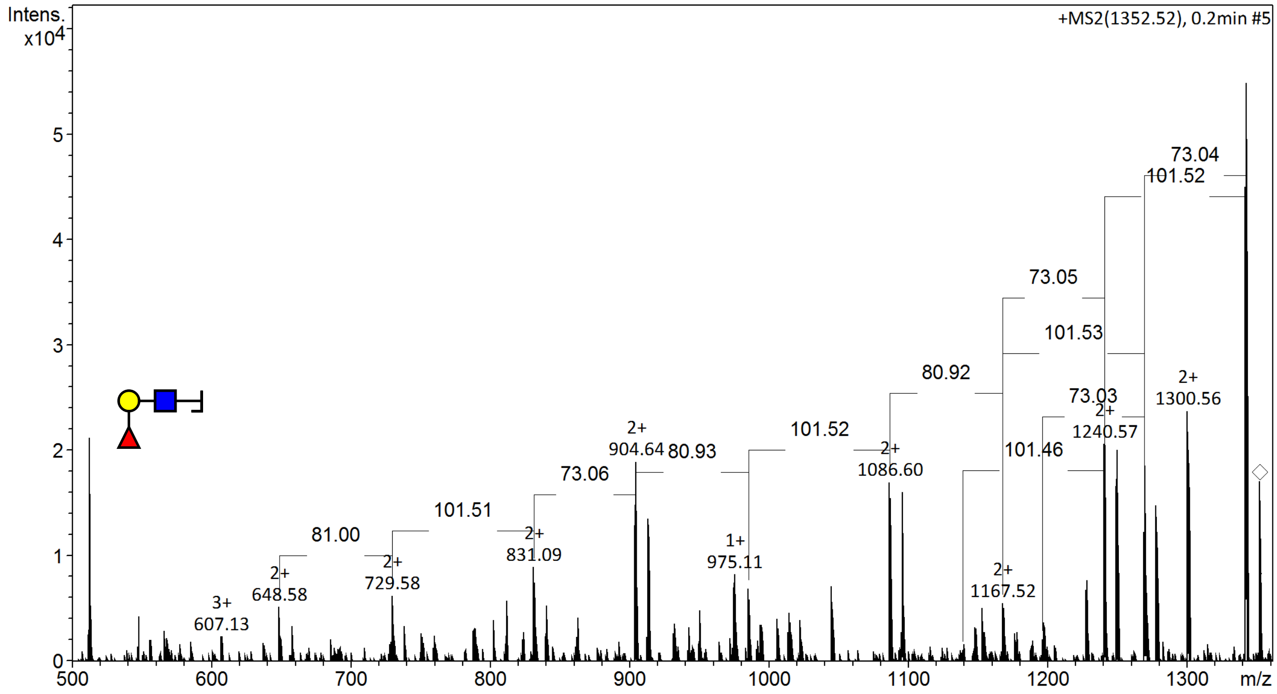

Bruker Compass DataAnalysis 4.2 printed: 12/23/2015 2:47:26 PM by: cbch Page 1 of 1

Figure S11: ESI-MS^2 of divalent N-glycan **23** in presence of 300 μM $CaCl_2$. Mass peak at m/z = 512 is indicative for H-type antigen.

Table S1: Data Collection and Refinement Statistics for LecB$_{PA14}$ structures

**Data-collection statistics**																
**Complex**	**Native**				**Me-α-L-Fuc (2)**				**Man-α1,3-Man (11)**				**Lewis^a (12)**			
Crystallization conditions	22% Peg8K, 0.2 M AMSO$_4$, 0.1 M Tris pH 8.5				26% Peg8K, 1 M AMSO$_4$, 0.1M Citric acid pH 3.8				24% Peg8K, 0.2 M AMSO$_4$, 0.1M Tris pH 8.5				22% Peg8K, 0.2 M AMSO$_4$, 0.1M Tris pH 8.5			
cryoprotectant	10% glycerol added				none				10% glycerol added				Increase Peg8K to 30%			
Beamline ESRF	BM30A				BM30A				ID23-1				BM30A			
Wavelength (Å)	0.92053				0.92053				0.8726				0.98021			
Spacegroup	P2$_1$				P2$_1$2$_1$2$_1$				P2$_1$				P2$_1$			
Unit cell: a,b,c (Å) α,β,γ (°)	52.9 49.6 75.5<br>90.0 93.4 90.0				52.5 65.6 109.0<br>90.0 90.0 90.0				53.0 49.8 75.3<br>90.0 93.3 90.0				53.0 63.8 64.5<br>90.0 91.2 90.0			
Resolution (outer shell), (Å)	41.42-1.7 (1.73-1.70)				37.84-1.55 (1.58-1.55)				44.52-1.4 (1.42-1.40)				27.82-1.50 (1.53-1.50)			
Measured/ Unique reflections	138777/43091				352300/55470				242799/76843				237721/66333			
Average multiplicity	3.2 (3.2)				6.4 (4.6)				3.2 (2.4)				3.6 (2.7)			
R_{merge}	0.079 (0.295)				0.067 (0.392)				0.037 (0.178)				0.028 (0.147)			
Completeness (%)	99.7 (99.0)				99.9 (98.5)				99.4 (94.9)				96.5 (72.4)			
Mean I / σI	10.1 (3.4)				20.9 (3.4)				21 (5.4)				26.9 (5.6)			
CC1/2	0.992 (0.831)				0.999 (0.867)				0.999 (0.931)				0.999 (0.959)			
R_{cryst}/ R_{free}	15.7/19.7				12.8/15.3				12.2/16.3				13.0/15.5			
nb reflections/free reflections	40973/2104				52637/2763				72808/4016				62930/3384			
R_{msd} bonds, (Å)	0.017				0.015				0.017				0.015			
R_{msd} angles (°)	1.60				1.64				1.80				1.71			
Rmsd chiral (Å^3)	0.099				0.10				0.113				0.095			
Chain	A	B	C	D	A	B	C	D	A	B	C	D	A	B	C	D
Protein atoms<br>Bfac (Å^2)	835<br>7.6	831<br>7.3	844<br>5.2	838<br>5.6	838<br>7.6	838<br>7.6	817<br>8.0	817<br>8.0	843<br>9.3	843<br>8.8	846<br>7.3	828<br>7.7	852<br>10.3	843<br>10.2	844<br>8.9	839<br>8.7
Calcium<br>Bfac (Å^2)	2<br>13.1	2<br>13.4	2<br>4.4	2<br>5.2	2<br>5.5	2<br>5.9	2<br>6.6	2<br>6.8	2<br>8.9	2<br>9.0	2<br>5.3	2<br>5.6	2<br>9.9	2.<br>8.4	2<br>6.5	2<br>7.1
Ligand atoms<br>Bfac (Å^2)	6<br>17.0	6<br>14.6	6<br>6.7	6<br>5.4	12<br>6.4	12<br>7.0	12<br>7.0	12<br>7.2	11<br>15.4	23<br>16.0	23<br>15.0	23<br>14.6	45<br>20.3	36<br>17.3	47<br>15.7	47<br>18.0
Water molecules<br>Bfac (Å^2)	174<br>20.0	154<br>18.3	169<br>18.6	151<br>19.5	218<br>20.7	205<br>20.1	188<br>21.6	147<br>22.0	176<br>22.0	177<br>22.4	185<br>22.3	143<br>22.2	221<br>24.9	219<br>24.5	232<br>23.4	191<br>23.4
Ramachandran plot :<br>Allowed /Favored /Outliers (%)	100 / 97.1 / 0				100 / 97.2 / 0				99.6 / 97.1 / 2				100 / 97.1 / 0			
PDBcode	5A6Q				5A6X				5A6Y				5A6Z			
